# Recent advances in the *ab initio* theory of solid-state defect qubits

**DOI:** 10.1515/nanoph-2022-0723

**Published:** 2023-02-01

**Authors:** Ádám Gali

**Affiliations:** Wigner Research Centre for Physics, PO. Box 49, Budapest H-1525, Hungary; Department of Atomic Physics, Institute of Physics, Budapest University of Technology and Economics, Műegyetem rakpart 3., Budapest H-1111, Hungary

**Keywords:** density functional theory, many-body perturbation theory, solid-state defect qubits, spin coherence time, spin relaxation time, theoretical spectroscopy

## Abstract

Solid-state defects acting as single photon sources and quantum bits are leading contenders in quantum technologies. Despite great efforts, not all the properties and behaviours of the presently known solid-state defect quantum bits are understood. Furthermore, various quantum technologies require novel solutions, thus new solid-state defect quantum bits should be explored to this end. These issues call to develop *ab initio* methods which accurately yield the key parameters of solid-state defect quantum bits and vastly accelerate the identification of novel ones for a target quantum technology application. In this review, we describe recent developments in the field including the calculation of excited states with quantum mechanical forces, treatment of spatially extended wavefunctions in supercell models, methods for temperature-dependent Herzberg–Teller fluorescence spectrum and photo-ionisation thresholds, accurate calculation of magneto-optical parameters of defects consisting of heavy atoms, as well as spin-phonon interaction responsible for temperature dependence of the longitudonal spin relaxation *T*_1_ time and magneto-optical parameters, and finally the calculation of spin dephasing and spin-echo times. We highlight breakthroughs including the description of effective-mass like excited states of deep defects and understanding the leading microscopic effect in the spin-relaxation of isolated nitrogen-vacancy centre in diamond.

## 1 Introduction

Quantum information is physical [1]. Solid-state defect spins are a conceivable platform to realize the elementary unit of quantum information, i.e., quantum bits or qubits [2]. Two prototypical representatives are the phosphorus donor (P-donor) in silicon and the nitrogen-vacancy (NV) centre in diamond. From electronic structure point of view, these two defects reside the opposite sides of the spectrum: the P-donor can be described by the so-called effective mass state with hydrogen-like Rydberg series of excitation energies split from the conduction band of silicon, which are weakly localized wavefunctions, whereas the carbon dangling bonds in NV centre create strongly localized orbitals with deep levels in the fundamental band gap of diamond. Kane proposed to apply P-donor spins as a qubit [3]. However, the read-out of the single spin in a controlled fashion had been great challenge for relatively long time that could be realized in a single-electron transistor device operating at hundreds of millikelvin temperatures [4]. The read-out of the single spin of diamond NV centre has been realised optically, i.e., optically detected magnetic resonance (ODMR), which was the first optically read single defect spin in a solid [5]. In this case, the readout and initialization of the electron spin of diamond NV centre could be readily carried out by optical means at room temperature. Recently, the single electron spin electrical read-out via photo-ionised electrons and holes has been realised for diamond NV centre which is a hybrid scheme: photo-excitation is required for creating spin-dependent photocurrent from a single NV defect and optical initialisation of the spin, and then the spin-dependent photocurrent is observed to read the electron spin state [6, 7]. The coherent manipulation of these electron spins were realised electron spin resonance techniques [8, 9]. The coherent control and readout of single defect spins define the underlying defects as quantum defects, and the quantum defect with its host material can be called as a quantum-coherent material.

Since the discovery and realisation of these quantum defects an intense research has begun to seek alternative solid-state defect spins both in the experimental and theoretical fronts, which might have favourable properties for certain quantum technology applications [10, 11]. Recently, data mining techniques with machine learning algorithms have been spread at the theoretical fronts. The data mining can be approached either towards the host materials [12–14] or to extend this with creating defect structures and calculating their key qubit parameters [15, 16]. In these approaches, there are assumptions for selection of materials and defects that might be too restrictive and might lead to overlooking important candidates. For instance, it was assumed that the host materials should have wide band gap with low density of nuclear spins, at least, for the defect qubits alike diamond NV centre [10, 12]. However, certain quantum technology applications such as quantum communication does not require room temperature operation, and small-band-gap silicon has become a promising platform to realize spin-to-photon interface with quantum memory [11, 17], [18], [19], [20], [21], [22], [23]. One of the most promising platforms to host qubits in two-dimensional (2D) materials is hexagonal boron nitride (hBN) which has 100% nuclear spin abundance but the coherence times of defect spins can be well extended with using good control of these nuclear spins [24–27]. Certainly, the selection criteria can be changed based on these recent findings. Nevertheless, quantum-coherent materials can only be interpreted together with their defect qubits, thus selection of host materials should be followed by finding defects for which the electron spin can be initialised and read-out with sufficiently long coherence times. Calculation of the coherence times for any hypothetical defects, e.g., Ref. [13], could lead to misleading results in certain cases where the spin density distribution and so the strongest hyperfine interaction between the electron spin and nearest nuclear spins characteristic for the actual defect would strongly affect its electron spin’s coherence time [24]. The automatically generated defects are often selected from the thermodynamically most stable ones [15, 16]. It has been found that this selection may omit very important complex defects realizing qubits, e.g., G-centre in silicon [28], which is one of most promising qubit candidates in silicon. Furthermore, it has been shown for diamond NV-centre [29] that the strongly coupled electron-phonon states are inevitable for understanding the optical spin-polarisation of its electron spin. These polaronic states are also usually ignored in these databases. These examples clearly call for improving the *ab initio* magneto-optical spectroscopy methods, in order to increase the credibility and prediction power of these databases.

*Ab initio* methods have significantly contributed to understanding and control of diamond NV qubits and exploration of alternative quantum defects which was summarized in a recent review paper [30]. In that review paper, the diamond NV qubit is thoroughly described including the electronic structure and polaronic solutions and spectra within Jahn–Teller theories, and the desiderata of the target of computations and the developed computational methods are summarized in detail which are not perpetuated here. We assume that the readers are aware of the basic description of diamond NV-like qubits and the previously implemented methods to compute their magneto-optical properties which are the basis of further developments.

For instance, new findings have been reported for the very basic property of this qubit such as the spin-lattice relaxation (*T*_1_) time [31] which apparently demonstrates that even the most studied diamond NV-centre has been not fully understood to date. It is an immediate quest to further advance *ab initio* methods, in order to accurately calculate the excited states together with quantum mechanical forces, the electron-phonon coupling and the basic magnetic parameters such as the zero-field-splitting between the electron spin’s levels (ZFS), or understanding the temperature dependence of these parameters and the spin-lattice relaxation (*T*_1_) time, and the coherence time (*T*_2_) also as a function of spin bath around the target defect qubit. This paper provides a comprehensive review about recent years *ab initio* developments on solid-state defect spin qubits along these directions.

We illustrate the advances of computational methods on various defects in solids: we shall discuss (i) deep defects such as NV centre, silicon-vacancy centre and nickel-vacancy centre in diamond, divacancy and vanadium centres in silicon carbide, and boron-vacancy in two-dimensional hexagonal boron nitride; (ii) the shallow excited states of deep defects such as the neutral silicon-vacancy centre in diamond and various interstitial defects in silicon; (iii) and shallow donors in silicon.

## 2 Computational methods

The *ab initio* investigation of solid-state defect qubits alike diamond NV-centre or silicon P-donor requires full and accurate description of the host material and the embedded isolated defect. The photostability of the quantum defects depends on the ionisation threshold energies, therefore it is critical that the crystalline bands and the resonant or localized defect states are computed at equal footing. Green-function methods are principally ideal to represent the topology of the problem, i.e., the embedded defect in a perfect solid. However, practical implementations of Green-function methods suffer from the consistent calculation of quantum mechanical forces which is required to calculate the ionic coordinates of the given point defect. As the geometry of the defect is highly decisive in their magneto-optical properties, the supercell method is most often employed to model the quantum defects which methodology readily offers the calculation of quantum mechanical forces based on the Hellman-Feynman theorem [30]. In this review article, we focus on those method developments and implementations which work within this formalism because the supercell formalism guarantees the simultaneously accurate calculation of the ionisation threshold and intra-defect optical transition energies. The ground state properties are typically calculated by means of Kohn-Sham density functional theory (KS DFT) [32], which can be a starting point for the calculation of excited states. A natural choice for the basis set for supercell formalism is the plane-wave basis which is combined by pseudopotential or projector augmentation wave (PAW) methods (see Ref. [33] and references therein). The computation methods of the ground state thermodynamic properties of point defects in solids within this formalism was already described in detail in the literature (e.g., Ref. [34]) that can be applied to the specific quantum defects [35]. In short, the quantum defect’s local properties and associated parameters are computed within plane wave supercell KS DFT methods, and implementation based on these methods or relied on the parameters obtained by these methods will be discussed in the review paper.

## 3 Method developments and results

In this section, we collect recent developments on *ab initio* calculation of properties of quantum defects. We start with the treatment of the excited states and geometries, and then we continue with the discussion of the ionisation threshold energies with the photo-ionisation spectrum, the supercell treatment of shallow defect states, the zero-field-splitting parameters, hyperfine parameters and gyromagnetic tensor, and the spin-lattice relaxation *T*_1_ times as well as the spin coherence *T*_2_ times. Although, it is unconventional to start the discussion with the excited states prior the description of finite size effects of supercells we decided to do so because we shall consider the finite size effects of shallow excited states in the section of supercell modelling of defects which requires description about the calculation of excited states. We shall also show that accurate description of the excited states is needed to calculate some ground state related magnetic parameters such as the zero-field splitting parameter.

### 3.1 Excited states

#### 3.1.1 Following the topology of the problem: DFT + CI multiscale methods

**Figure 1:**
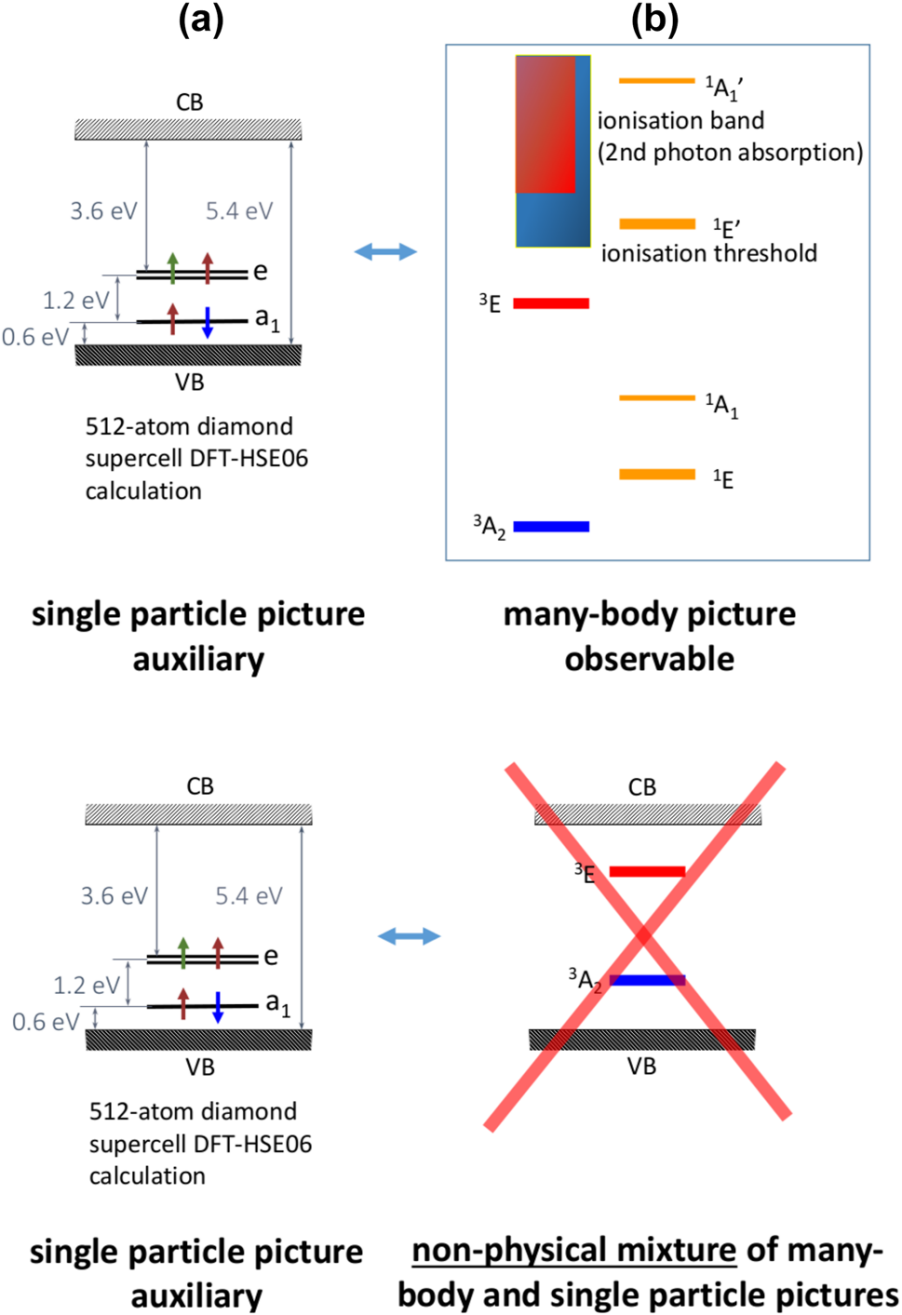
Defect levels diagramme. (a) Single-particle scheme of the electronic structure of diamond NV centre. The spin-polarisation between spin-up and spin-down electrons in the Kohn–Sham density functional theory results in different levels for spin-up and spin-down orbitals which are not depicted here but rather their average. VB and CB label valence band and conduction band, respectively. The fundamental band gap of diamond is 5.4 eV. (b) Many-body level structure of diamond NV centre. The blue and red shaded areas show the ionisation bands excited from the ^3^*A*_2_ and ^3^*E* states, respectively. In the bottom row the non-physical picture is shown which often appears in many scientific papers.

The accurate calculation of the excited states is still under extensive research. The popular many-body perturbation method, GW + BSE (see Ref. [36] and references therein), fails for the highly correlated singlet states of the diamond NV centre [37, 38]. The highly correlated states may be recognized of which many-body electronic wavefunction (Ψ) can be genuinely described by two or more Slater-determinants with significant weights. This may be quantified with the function of one-particle density matrix $\tilde{\rho }$. This can be defined as ${\tilde{\rho }}_{ij}=\left\langle \Psi |a_{i}^{†}a_{j}|\Psi \right\rangle$ with the creation and annihilation operators of single electrons *i* and *j*, respectively. The degree of correlated electronic state (Λ) is then

(1) $$\Lambda =\mathrm{Tr}\left(\tilde{\rho }-{\tilde{\rho }}^{2}\right)=\mathrm{Tr}\;\left(\tilde{\rho }\right)-\mathrm{Tr}\left({\tilde{\rho }}^{2}\right)\text{,}$$

as if and only if the Ψ can be described as a single Fock-state when $\tilde{\rho }={\tilde{\rho }}^{2}$. Those cases are in particular pathological in which double-excitation Slater-determinants appear with relatively strong contribution (i.e., $a_{i}^{†}a_{j}^{†}a_{k}a_{l}|\Psi_{\text{GS}}〉$ with the ground state many-body wavefunction Ψ_GS_) which is the case for the so-called ^1^*A*_1_ state of the diamond NV centre (see Figure 1) which plays an important role in the optical spin-polarisation and read-out processes (see Ref. [30] and references therein).

In KS DFT theory, these many-body states cannot be captured by the known exchange-correlation potentials (*V*_xc_); however, it provides a very good description for the host materials and simple defect states, i.e., for which Λ = 0. In general, the bands of the host crystal can be well described by KS DFT method whereas the strong Coulomb interaction between localized defect orbitals confined to a small place, e.g., around a vacancy, represents a problem for KS DFT method. The topology of the problem implies a method in which the Coulomb interaction between the localized defect orbitals is directly calculated, called configurational interaction (CI), which are in contact with the bath of itinerant electrons that can be treated by KS DFT. This can be considered as a multiscale problem where the interaction between electrons are calculated with different precisions in a single system. As we will see below the challenges are to define the set of orbitals, often called “active space”, for which the precise method should be applied (here, CI) and to find an interface between the levels of different approaches, here DFT and CI, which can produce self-consistent results. Interfacing different levels of theory in a single system is a common problem for all the multiscale methods.

By following the above mentioned topology of the problem, the many-body Hamiltonian may be described as

(2) $$\hat{H}=\overset{A}{\underset{ij}{\sum }}t_{ij}^{\text{eff}}a_{i}^{†}a_{j}+\frac{1}{2}\overset{A}{\underset{\mathrm{ijkl}}{\sum }}v_{\mathrm{ijkl}}^{\text{eff}}a_{i}^{†}a_{j}^{†}a_{l}a_{k}\text{,}$$

where *v*^eff^ is the partially screened Coulomb-interaction $\left(W_{0}^{R}\right)$ and the *ijkl* KS wavefunctions are within the active space *A*. Partial screening is computed from all the KS wavefunctions except for the set of KS wavefunctions within *A*. The definition of *v*^eff^ includes contributions to the Hartree and exchange correlation energies that are also included in the KS DFT calculations for the whole solid. Exactly this is the point where an interface between DFT and CI methods should be developed which can be considered as a typical problem for all the related electronic structure embedding schemes called double counting (dc) error. Therefore, the KS DFT Hamiltonian ${\hat{H}}_{ij}^{\text{KS}}$, occurring in the first term in Eq. (2), requires double counting correction $t_{ij}^{\text{dc}}$, i.e.,

(3) $$t_{ij}^{\text{eff}}={\hat{H}}_{ij}^{\text{KS}}-t_{ij}^{\text{dc}}\text{.}$$

The methodology was first implemented by Bockstedte and co-workers within VASP code [39, 40] which was called DFT + CI-cRPA method [41]. Here, CI refers to the configurational interaction between the electrons in the active space whereas constrained random phase approximation was applied to compute $W_{0}^{R}$ [41]. In that implementation, Heyd–Scuzeria–Ernzerhof (HSE06) [42] functional was applied which includes a quarter of Fock-exchange (*α* = 25%) in the KS potential for the KS orbitals. To calculate $t_{ij}^{\text{dc}}$, a heuristic approach was applied, i.e., the quarter of Fock-exchange was used for the electrons in the active space. This approach resulted in a very good agreement between the computed spectra and the spectra derived from experiments for diamond NV centre and silicon carbide divacancy centres [41]. Later this treatment was also implemented to the Quantum Espresso software package [43] and the CI part of the Hamiltonian was also interfaced with a code running on quantum computers [44]. The quality of present quantum computers precludes to achieve accurate results [44], thus the results achieved by classical computers will be summarized below. The DFT + CI multiscale method was then rephrased to quantum embedding or quantum defect embedding method but it is essentially the same with the originally invented CI-cRPA method with the caveat that the latter implementation used a full Hartree–Fock dc correction (HFDC), i.e., the exchange term was not scaled by the fraction of the applied hybrid DFT functional [41, 44]. Ma and co-workers also went beyond RPA (b-RPA) by taking the exchange-correlation effects into account in the calculation of $W_{0}^{R}$, and they applied it to diamond NV centre [44]. It was found that b-RPA screening substantially modifies only the ^1^*A*_1_ level referenced to the ground state energy with respect to the results within RPA screening approach (see Table 1). The origin of this effect is not explained and well understood. Pfäffle and co-workers implemented a semiempirical DFT + CI method [45]: they basically applied HFDC to the DFT or GW quasi-particle (GWQP) levels in Eq. (3). The screening was taken from the bulk diamond with an analytical formula developed for semiconductors which provided the accuracy of the RPA method [46]. Resta found that the space-dependent dielectric screening only varies within the range of nearest-neighbour distance of ions in semiconductors, if the origin is chosen to an ion in the crystal, with a well-defined function and then it approaches the dielectric constant of the material, *ɛ*(0) [46]. Taking the host material’s dielectric screening function for a defect might be too inaccurate, in particular, for defects which contain vacancy as no ion would “modulate” the dielectric constant of the host material in the region of vacancy. Therefore, Pfäffle and co-workers introduced a semi-empirical formula to modify the dielectric screening function near the vacancy [45] of diamond NV defect which they called “masked” solution. It is unclear how the parameter in that formula was picked up. With the “masked” solution for the diamond NV centre they obtained triplet optical transition close to the DFT + CI-cRPA solution [41] whereas the energy gap between the singlets closely follows that of b-RPA screening with HFDC [44]. We note that this method was further applied to the neutral and positively charged NV defect in diamond. The optical spectrum is known for the neutral NV defect in diamond [47, 48] for which the ZPL energy results in 2.156 eV between ^2^*E* ground state and ^2^*A*_2_ excited state. Although, the order of the many-body levels is consistent with the experimental data by the semi-empirical DFT + CI method but the calculated energy gap for ^2^*E* ↔ ^2^*A*_2_ transition is at 1.65 eV which is disappointingly low. It is yet unclear what is the origin behind the discrepancy.

**Table 1:** The calculated electronic structure of diamond NV centre with using different types of DFT + CI embedding methods. The ground state is ^3^*A*_2_ and all the levels are referenced to it with fixed ionic coordinates of the electronic ground state. The type of DFT functional is given: the DDH functional provides *α* = 17.8% Fock-exchange for diamond [44]. In HSE06 functional [42] *α* = 25% was applied. The applied approximation for calculating $W_{0}^{R}$ is either cRPA or goes beyond (b-RPA). Finally, the dc correction is either Hartree–Fock type (HFDC), or scaled with *α* in the DFT functional (*α*FDC). The exact dc (EDC) correction is applied together with quasiparticle (QP) levels within self-consistent GW method (see text). The experimental data are only estimates from PL spectrum and rate modelling of the observed decay rates (see Ref. [30] and references therein).

NV	HSE06 [41]	HSE06 [49]	DDH [44]	DDH [44]	GWQP [50]	GWQP [50]	Exp.
	cRPA	cRPA	cRPA	b-RPA	cRPA	cRPA	
	*α*FDC	HFDC	HFDC	HFDC	HFDC	EDC	
^1^ *E*	0.49	0.49	0.476	0.561	0.375	0.463	∼0.40
^1^ *A*_1_	1.41	1.39	1.376	1.759	1.150	1.270	∼1.55
^3^ *E*	2.02	2.06	1.921	2.001	1.324	2.152	∼2.15

In any embedding methods it is a crucial question how to select the active space orbitals. The in-gap defect states are *per se* localized, therefore, they are naturally involved in the active space. However, defect states *resonant* with the valence band or conduction band may exist which are coupled to the Bloch-states of the host material, thus they do not appear as sharp resonances but are rather broadened. In practice, the coupling strength and the width of broadening depend on the size of supercell and k-point sampling of the Brillouin-zone for the given supercell. For instance, it was found that whereas the lowest energy spectrum of diamond NV centre may converge relatively well for the minimal active space taken from the in-gap defect orbitals but this treatment leads to a serious inaccuracy for the isovalent silicon carbide divacancy defect for which explicit involvement of the resonant states in the valence band is necessary to achieve converged results [41]. This result clearly demonstrated that simplified Hubbard-U model with using only the in-gap defect orbitals (e.g., Ref. [38]) is generally insufficient for accurate description of the many-body electronic structure of quantum defects. In practice, an energy region of about 3 eV around the Fermi-level was used to pick-up the states for the active space with 512 and 576-atom supercell models in the original study [41]. In later studies, the choice of the active space was further investigated [49, 50]. Muechler and co-workers also implemented the DFT + CI-cRPA method to the VASP code but they first constructed Wannier wavefunctions [51] with preserving the position of the in-gap defect levels which could maximally localize the defect wavefunctions including those that are resonant with the host bands, and then the Wannier orbitals were applied in Eq. (2). With this treatment the results upon the number of orbitals in the active space can rapidly converge [49]. Another possibility is to pick-up the states based on localization of the KS orbitals that can be simply quantified as $L_{V}\left(\psi_{i}^{\text{KS}}\right)=\int_{V\subseteq \Omega }{\left|\psi_{i}^{\text{KS}}\right|}^{2}dV$, where *V* is a chosen volume including the defect, smaller than the supercell volume Ω. It was found that *L*_*V*_ = 5% is needed to obtain convergent results for the notorious ^3^*E* state which corresponds to about 40 (30) states in the active space for 216-atom (512-atom) supercell model [50]. The ^3^*E* state is notorious in terms of *L*_*V*_ because *a*_1_ hole orbital is involved in this state of which level lies close to the valence band which leads to exchange-correlation coupling to resonant states in the valence band. This is not the case for the ^1^*E* and ^1^*A*_1_ levels with very little contributions from the *a*_1_ hole orbital, so they converge fast in this regard.

Another critical question in the embedding methods is the treatment of double counting correction which has been briefly considered above. In Hartree–Fock methods, the double counting correction can be readily derived and it is applied routinely in quantum chemistry. However, DFT is applied to calculate the electronic structure of the ground state in the supercell modeling of defects, and there is no theoretical rationale to apply HFDC in this case. By applying hybrid DFT functionals, Fock-exchange is employed to KS orbitals. By assuming a tiny contribution of the semilocal exchange-correlation in dc correction, one may apply *α*FDC meaning that the corresponding fraction of the Fock-exchange for the active space orbitals is employed. This heuristic treatment may follow the idea of the DFT + U treatment in which U is an orbital-dependent on-site correction, and that is scaled by *α* when hybrid DFT is applied [52]. However, hybrid DFT is still a functional of the electron density and not the many-body wavefunction, thus this heuristic treatment strictly cannot be justified.

Recently, Sheng and co-workers derived the dc correction for KS DFT with using the Green-function approach [50] which might be motivated by the success of interfacing KS DFT and dynamical mean field theory (DMFT) with the same approach [53]. They assumed that the non-diagonal terms of the self-energy coupling the active space and the environment is negligible, and static approximation is used for the *G*_0_ and *W*_0_. This results in

(4) $$t^{\text{dc}}=V_{\text{xc}}+W_{0}^{R}\rho_{A}-iG_{0}^{R}W_{0}\text{,}$$

where *ρ*_*A*_ is the density matrix of the electrons within the active space, and *G*_0_ and *W*_0_ are computed at the quasiparticle energies (*ϵ*^QP^) of these electrons [50]. The exact dc correction is called here EDC in which the quasiparticle (QP) levels are solved self-consistently with calculating ∑_xc_ self-energy within GW method as $\epsilon_{i}^{\text{QP}}=\epsilon_{i}^{\text{KS}}+\left\langle \psi_{i}^{\text{KS}}|\sum_{\text{xc}}\left(\epsilon_{i}^{\text{QP}}\right)-V_{\text{xc}}|\psi_{i}^{\text{KS}}\right\rangle$. This makes the *ϵ*^QP^ results almost independent from the applied DFT functional within 0.1 eV, it was either the semilocal Perdew–Burke–Ernzerhof (PBE) [54] or the dielectric-dependent hybrid (DDH) [55] functional. The results are summarized for diamond NV centre in Table 1 where they applied both HFDC and EDC corrections. They reported extremely low energy for ^3^*E* state with HFDC which was not explained in detail [50]. The EDC results are quite comparable to the genuine CI-cRPA result with the heuristic *α*FDC correction, nevertheless, the good agreement might be specific to diamond NV centre.

Sheng and co-workers applied the EDC method also to the neutral silicon-vacancy (SiV) defect and their sister group-IV vacancy defects in diamond [50]. The ground state of the defect is ^3^*A*_2*g*_ which is a similar wavefunction to that of diamond NV centre. However, the excited state of the neutral SiV defect is highly complex with leaving a hole in each double degenerate orbital which results in three triplet states (^3^*A*_2*u*_, ^3^*E*_*u*_, ^3^*A*_1*u*_). These triplet states are highly correlated and they are also coupled by phonons via product Jahn–Teller interaction [56, 57]. According to that study, the product Jahn–Teller interaction leads to a strong ionic relaxation upon excitation (≈0.3 eV), so the vertical excitation energy of the optically allowed ^3^*E*_*u*_ state should lie at around 1.6 eV above the ground state level. The EDC calculation yielded disappointedly high energy for ^3^*E*_*u*_ at 2.161 eV when *L*_*V*_ = 5% was applied [50]. We note that the calculated excitation energies for the triplet excited states do not converge monotonously with decreasing the value of *L*_*V*_ and their values are close to the desired energies with setting *L*_*V*_ = 20%. It is unclear yet what is the origin of this behaviour.

Despite the remaining issues with the DFT + CI multiscale methods, this approach is very promising to calculate the highly correlated defect states within supercell formalism. The very important next step is to derive the quantum mechanical forces which is then can be used to reoptimise the geometry of the ground state and the excited states. This is required to calculate the characteristic zero-phonon-line energies and Debye–Waller factors of the defects which are key parameters of quantum emitters. The big challenge here is that the original derivation of the EDC method assumed that the off-diagonal term of the self-energy coupling the active space and the environment can be neglected but once the forces are considered one has to take into account the change of the character of the underlying KS wavefunction self-consistently with the self-energy through the coupling between the active space and the environment. In general, it is a known problem in quantum chemistry CI methods that the final results may depend on the initial single particle wavefunctions if restricted active space is applied and the underlying single particle wavefunctions are fixed at the electronic ground state manifold. The DFT + CI multiscale methods face to the same problem, principally.

#### 3.1.2 Density matrix renormalization group methods: an alternative multiscale method

Density matrix renormalization group (DMRG) was originally developed to describe one-dimensional quantum models in solid-state physics with local interactions. The underlying mathematical framework, however, is not restricted to models studied in condensed matter physics or applications to molecular clusters but among many others, it can be also used to study nuclear shell models, particles in confined potential, or problems in the relativistic domain. The success of these developments relies on the efficient factorization of interactions and the optimization of the DMRG network topologies based on concepts of quantum information theory leading to tremendous reduction in computational costs (see Ref. [58] and references therein). In particular, the factorization for the many-body wavefunction Ψ with *L* spatial orbitals reads as

(5) $$|\Psi 〉=\underset{\left(n\right)}{\sum }C^{\left(n\right)}\overset{L}{\underset{i=1}{\prod }}{\left(a_{i↑}^{†}\right)}^{n_{i↑}}{\left(a_{i↓}^{†}\right)}^{n_{i↓}}|0〉$$

with

(6) $$C^{\left(n\right)}=\overset{L}{\underset{i=1}{\prod }}A_{i}^{\left(n_{i↑}n_{i↓}\right)}\text{,}$$

where now the spin state (*σ*) is explicitly written by arrows and *n* = (*n*_1↑_*n*_1↓_*n*_2↑_*n*_2↓_ … *n*_*L*↑_*n*_*L*↓_) where *n*_*iσ*_ ∈ {0, 1}. The components of the state specific *C* tensor increase exponentially with system size *L* scaling as 2^2*L*^ which becomes untractable for few hundreds of electrons. However, the dimension of the matrix product states *A*_*i*_ can be optimized in DMRG approach, $A_{i}^{\text{DMRG}}$, truncated to a fixed manageable bond dimension, *M*, that is, $\dim\left(A_{i}^{\text{DMRG}}\right)\leq \left[M,M\right]$, i.e., *M* × *M* tensor. Increasing *M*, the precision of the approximation is well-controlled approaching variationally the exact solution. In the DMRG protocol, the matrix product state matrices are locally optimized and truncated by minimizing the discarded entanglement between the left and right neighbouring blocks of the matrix product state chain, obtained from the reduced density matrix of the block. The algorithm iterates through the matrix product state chain in a sequential order back and forth until reaching convergence.

In short, application of DMRG method on electron-nuclei systems can be considered as a special wavefunction method that can be used to accurately calculate the static correlation between electrons. Barcza and co-workers extended this method to interface with DFT calculations of quantum defects [58]. In this post-DFT method, the Coulomb integrals of the KS orbitals are directly taken from the supercell DFT calculation in the electronic ground state manifold which is post-processed by DMRG algorithms. Despite the advantage of DMRG method, hundreds of KS orbitals cannot be directly treated owing to the computational costs. Therefore, an optimal selection of orbitals with tractable size is needed which is responsible for the strong static correlations. This was carried by the complete active space (CAS) self-consistent field method which is well-known in quantum chemistry (see Ref. [58] and references therein). The CAS method classifies the set of orbitals to three categories; that is, the so-called core and virtual orbitals are frozen to the mean field level and filled with two and zero electrons, respectively. The third class comprises of the so-called active space orbitals which are populated with the rest of electrons minimizing the energy. Accordingly, the virtual orbitals does not play any role in the corresponding CAS Hamiltonian, whereas the core electrons affect the electrons of the active space through the Coulomb interactions, that is, the Hamiltonian of the active space reads

(7) $$\begin{array}{ll} {\hat{H}}^{\text{CAS}} & =E^{\text{nuc}}+E^{\text{core}}+\underset{ij}{\sum }t_{ij}^{\text{CAS}}a_{i}^{†}a_{j}\\ & \;+\frac{1}{2}\underset{\mathrm{ijkl}}{\sum }V_{\mathrm{ijkl}}a_{i}^{†}a_{j}^{†}a_{k}a_{l}\text{.} \end{array}$$

The one-electron integrals of the CAS space, $t_{ij}^{\text{CAS}}$, describe not only the kinetic energy of the electrons in the active space and their attraction to nuclei but also their interaction with the core electrons. Describing the electrons in the active space with the DMRG method, which treats exactly the electron exchange, the one-electron interactions are written as

(8) $$t_{ij}^{\text{CAS}}=t_{ij}+\frac{1}{2}\underset{c}{\sum }\left(2V_{\mathrm{iccj}}-V_{\mathrm{icjc}}\right)$$

to treat the Coulombic effects of the frozen electrons on the active space orbitals. In other words, this is a multiscale method where the interface between the active space and the environment can be well managed within Hartree–Fock level. Here, the summation runs only on the indices of the core orbitals (*c*). Finally, the additional energy contribution of the core electrons is summed up in term *E*^core^, that is

(9) $$E^{\text{core}}=2\underset{c}{\sum }t_{cc}+\underset{cc^{\prime }}{\sum }\left(2V_{cc^{\prime }c^{\prime }c}-V_{cc^{\prime }cc^{\prime }}\right)\text{.}$$

In practice, the active space is restricted to the most important orbitals featuring strong correlation as was discussed for DFT + CI methods. Even though the method has limitations to provide correct description of dynamic correlations using the relatively small active space, it captures static correlations with high accuracy providing valuable insights into the low-lying energy spectrum and the essential structure and symmetry properties of the corresponding electronic eigenstates. Note also that, contrary to DFT + CI methods, the CAS Hamiltonian in Eq. (7) does not include the KS energies explicitly but only the KS orbitals by construction. Also, the absolute energies of the states computed from the CAS Hamiltonian are not trivially comparable with counterparts obtained on the DFT level of theory due to the different description of the exchange and correlation effects.

The methodology was first applied to the negatively charged boron-vacancy defect in hexagonal boron nitride (hBN) [59]. Previous *ab initio* calculations predicted this defect in hBN as a qubit [60] of which ODMR signal was confirmed in experiment [61] and has become a leading quantum defect in two-dimensional (2D) materials. This defect consists of three nitrogen dangling bonds which introduce four closeby levels in the fundamental band gap. Since the vacancy lobes are closely spaced with strong Coulomb interaction they form highly correlated states. The order of the many-body levels and the nature of the states were determined by DMRG method [62]. In the calculations about 50 KS orbitals were used in the active space selected by localization criterion. It was shown that the flake model with about 80 B and N atoms with hydrogen termination of the edge of the flake provides the same electronic structure as the periodic supercell model at DMRG level [58]. This makes it realistic to calculate the electronic structure of defects within the flake model by means of traditional quantum chemistry wavefunction methods [63, 64]. We note that the validity of the flake (molecular cluster) model could be specific to (planar) defects in hBN and may be not applied in general for defects in other 2D materials.

DMRG method was also applied to magnesium-vacancy (MgV) centre in diamond [65]. MgV centre was created by Mg implantation to diamond which has a unique photostability [66] which makes it an interesting quantum emitter. The PL signal was associated with the negatively charged MgV defect which has a similar electronic structure to that of SiV centre [65] with a caveat that the resonant *a*_2*u*_ level lies very close to the top of the valence band and the two degenerate *e*_*u*_ and *e*_*g*_ levels lie in the fundamental band gap. These states are localized on the vacancy lobes which create highly correlated many-body states. In particular, the neutral MgV has overwhelmly complicated electronic structure which needs wavefunction method such as DMRG.

Beside the optimization of the selection of the active space orbitals, a critical issue to take the dynamical correlation effects into account. The present Hartree–Fock treatment of the core orbitals [58] could be insufficient for many materials to achieve accurate electronic structure. In order to achieve an extremely accurate total energy of crystals, a coupled-cluster-single-double with perturbative triple [CCSD(T)] wavefunction approach is required [67]. It is likely that accurate low-energy excitation spectrum around the Fermi-level may be achieved at much lower complexity of wavefunction approaches than CCSD(T). One possible route of development is the implementation of more complex levels of wavefunction approaches than Hartree–Fock core and full CI active space model in the DMRG multiscale method, in order to converge towards highly accurate low-energy excitation spectrum. Another important issue, similarly to the DFT + CI methods, is to compute quantum mechanical forces acting on ions. The concept of the force calculation does exist for DMRG method that was already implemented for quantum chemistry codes (see Ref. [68] and references therein).

#### 3.1.3 Spin-flipping time-dependent density functional theory and BSE methods

Time-dependent DFT (TDDFT) based on KS-DFT in the kernel [69, 70] can be principally applied to calculate the low energy excitation spectrum of quantum defects [71, 72]. In order to achieve accurate results, the proper choice of the DFT functional is essential [71]. Unlike the present implementations of GW + BSE and post-DFT multiscale methods, TDDFT framework and implementations exist to calculate the quantum mechanical forces acting on ions in the electronic excited state. In a pioneer work it was shown (see Supplementary Materials in Ref. [73]) that the observed Stokes-shift of NV centre can be well reproduced by TDDFT calculation when the NV centre is embedded in the core of 1.4-nm sized nanodiamond. The optimized geometry by TDDFT method well reproduced the optimized geometry by the ΔSCF method [74] for diamond NV centre. This issue has been recently investigated for the supercell model of diamond NV centre and silicon carbide divacancy centres [75]. It was found that the optimized geometries, the adiabatic potential energy surfaces (APES) of the ^3^*E* state and the zero-phonon-line (ZPL) energies are very close to each other as obtained ΔSCF method and TDDFT method based on DDH functional (see Figure 4 in Ref. [75]). The reason behind this good agreement is that Λ ≈ 0 for ^3^*E* so it is a very good approximation to describe ^3^*E* state as promotion a single electron from *a*_1_ in-gap defect level to the *e* in-gap defect level in the spin minority channel which is exactly constructed by ΔSCF method.

In the recent years, spin-flipping TDDFT (sf-TDDFT) theory has been developed and applied to molecules [76, 77] which is based on the Casida equations [70] but the original equations are modified in order to calculate the states and energies associated with Δ*m*_*s*_ = ±1 spin transition. In principally, the supercell implementation of the sf-TDDFT can be applied to diamond NV centre to obtain the singlet ^1^*A*_1_ and ^1^*E* states with geometry optimization in these states that has not yet been achieved by other means. In the DFT + CI-cRPA calculations [41], it was already recognized for diamond NV centre that the ^1^*E* state contains Slater-determinants associated with symmetry breaking solutions, thus ^1^*E* state is dynamically distorted from the *C*_3*v*_ high symmetry geometry unlike the ^1^*A*_1_ state which stays in *C*_3*v*_ symmetry. Insights from group theory, ΔSCF and DFT + CI-cRPA calculations with electron-phonon Hamiltonian models made it possible to construct the absorption and emission spectra between the ^1^*A*_1_ and ^1^*E* states for NV centre [29]. Nevertheless, the construction of the absorption spectrum was not accurate as principally it could not use the true APES of ^1^*E* and ^1^*A*_1_ states. As a consequence, the sharp resonance in the absorption band at 170 meV above the diamond phonon bands was missing in the constructed absorption spectrum.

In a seminal work by Jin and co-workers [78], the singlet states of diamond NV centre were directly calculated by sf-TDDT including quantum mechanical forces. The *ab initio* APES could be calculated both for the ^1^*E* and ^1^*A*_1_. Although, the sf-TDDFT excitation does not involve the double excitation electronic configurations in ^1^*E* and ^1^*A*_1_ states but their contributions may influence their energy levels – higher than that by a DFT + CI method [50, 78] – but not significantly their optimized geometries. The previously developed model Hamiltonians based on ΔSCF calculations [29] have been confirmed by sf-TDDFT calculations [78]: the ^1^*E* state is strongly anharmonic whereas the ^1^*A*_1_ state shows an almost perfect parabolic APES but the effective phonon frequencies are higher than that for ^3^*A*_2_ ground state. On the other hand, the ^1^*A*_1_ state shows a slight anharmonicity due to its phonon coupling to the ^1^*E* state by the symmetry breaking *e* phonons. Taking this correction into account, the calculated absorption spectrum shows a perfect agreement with the observed absorption spectrum including the sharp vibration resonance at 170 meV [78]. It was found that the *e* phonons dominantly contribute to the absorption spectrum, in stark contrast to the optical spectra between the triplet states of the defect. The luminescence spectrum between singlets was not calculated in this study [78] which is a Herzberg–Teller optical transition [29], and the highly accurate calculation of the shape of the phonon sideband would need to solve the multi-mode Jahn–Teller problem [79].

Spin-flip BSE (sf-BSE) method can be basically also applied to calculate Λ ≫ 0 states. Here the original idea is that |Ψ_GS_⟩ of the system could be a close shell singlet but the low-energy singlet excited state might be a $a_{i}^{†}a_{j}^{†}a_{k}a_{l}|\Psi_{\text{GS}}〉$ type. This may be addressed by a high-spin reference state, e.g., $a_{i}^{†}a_{j}|\Psi_{\text{GS}}〉$ type of *S* = 1 shelving state, by flipping the spin state by *m*_*s*_ = −1 to reach |Ψ_GS_⟩ (negative excitation energy) or by raising with *m*_*s*_ = +1 to reach the excited state of $a_{i}^{†}a_{j}^{†}a_{k}a_{l}|\Psi_{\text{GS}}〉$ type (positive excitation energy). The sf-BSE spectrum can be calculated by ignoring the exchange terms in the BSE kernel. This methodology was first applied to atoms and molecules [80]. Parallel to this effort, Barker and Strubbe applied this method to diamond NV centre [81] with using PBE DFT functional as implemented in the Octopus code [82, 83]. By choosing *S* = 1 reference state raises the issue of spin-polarization within KS-DFT approach which naturally results in spin contamination error and an “artificial” gap between the occupied an unoccupied GWQP levels. Barker and Strubbe rather took the KS DFT energy levels instead of the GWQP levels in the BSE kernel as a workaround because PBE KS levels do not show this problem owing to the semilocal nature of the PBE functional. They picked up the ^3^*A*_2_ state as a reference state which is the ground state of diamond NV centre. The computed spectrum basically agreed with the previous GW + BSE result [37], i.e., the ^1^*A*_1_ level lies too low in the spectrum [81]. In order to calculate the ^1^*A*_1_ level correctly with sf-BSE method, the ^3^*E* reference state may be chosen which can access both single and double excitation Slater-determinants with respect to ^3^*A*_2_ ground state. Even though sf-BSE may work out for the singlet states accurately, the calculation of quantum mechanical forces should be implemented within GW + BSE and GW + sf-BSE methods to calculate the ZPL and Debye–Waller factor of the optical excitation spectrum of the defect qubits.

#### 3.1.4 Temperature broadening of optical excitation spectrum with BSE method

The many-body perturbation theory of electron-phonon coupled optical transition with non-equlibrium Green-functions has been developed in Ref. [84] which was further derived in Ref. [85]. They considered the adiabatic limit for the dipole matrix elements, while they retained dynamical effects only in transition energies [85]. In terms of the phonon-dependent optical dipole transition moments, it goes beyond the Huang–Rhys theory. The central equation is

(10) $$\begin{array}{ll} I^{\text{BSE}}\left(\omega ,T\right) & \propto \underset{\lambda ,\nu }{\sum }\frac{\partial^{2}|\Pi_{\lambda }{|}^{2}}{\partial x_{\nu }^{2}}f_{\lambda }^{<}\left[\delta \left(\omega -E_{\lambda }-\omega_{\nu }\right)\frac{n_{B}\left(\omega_{\nu },T\right)}{2\omega_{\nu }}\right.\\ & \;\left.+\delta \left(\omega -E_{\lambda }+\omega_{\nu }\right)\frac{1+n_{B}\left(\omega_{\nu },T\right)}{2\omega_{\nu }}\right]\text{,} \end{array}$$

where Π_*λ*_ are the exciton (*λ*) dipole matrix elements within BSE theory, and $f_{\lambda }^{<}$ are their occupations with *E*_*λ*_ energy; *x*_*ν*_ is the normal coordinate of phonon *μ* with the energy of *ω*_*ν*_. Here, $f_{\lambda }^{<}$ is non-vanishing only if the excitons are composed by transitions between bands occupied by excited electrons and holes, and the two Dirac *δ* in the square bracket correspond to the cases where an exciton recombines with the creation [*δ*(*ω* − *E*_*λ*_ + *ω*_*ν*_)] or annihilation [*δ*(*ω* − *E*_*λ*_ − *ω*_*ν*_)] of a phonon; *n*_*B*_ is the Bose–Einstein occupation function for phonon *ν* at temperature *T*. The no-phonon optical transition is given by

(11) $$I_{0}^{\text{BSE}}=\underset{\lambda }{\sum }|\Pi_{\lambda }{|}^{2}f_{\lambda }^{<}\delta \left(\omega -E_{\lambda }\right)\text{.}$$

In the usual implementation of BSE, it does not take into account the polaron shift. In other words, the no-phonon or zero-phonon line energy is calculated at the fix coordinate of the ground state from which the GW + BSE calculation starts. The theory was first applied to bulk hexagonal boron nitride which has indirect band gap, so $I_{0}^{\text{BSE}}=0$ (see Ref. [85]). Libbi and co-workers implemented the theory and applied it to the negatively charged boron-vacancy in hexagonal boron nitride [86]; the importance of defect was already mentioned in this review paper.

The observed fluorescence comes from a first-order forbidden transition between the ^3^*E*′′ excited state and ^3^$A_{2}^{\prime }$ ground state [62] which becomes only allowed by participation of phonons. It was suggested based on Δ*SCF* HSE06 calculations that symmetry distorting Jahn–Teller distortions could lead to optical transitions. While it was noted based on the comparison DFT HSE06 and DMRG methods that HSE06 Δ*SCF* method has a limitation in describing the APES of ^3^*E*′′ excited state, still the Huang–Rhys fluorescence spectrum was calculated with relatively good agreement with the overall fluorescence energy but the calculated phonon sideband seemed to be too wide when compared to experimental data (see Ref. [62] and references therein). The in-plane Jahn–Teller distortion resulted in about 193 meV energy gain compared to that of the high *D*_3*h*_ configuration. Further symmetry reduction and energy gain of 13 meV were also observed by HSE06 ΔSCF calculations by out-of-plane phonon modes [62]. The out-of-plane phonon modes are transformed as $A_{2}^{\prime \prime }$ and *E*′′.

Libbi and co-workers rather applied G_0_W_0_ method on PBE DFT calculations [86]. Then they calculated the non-equlibrium BSE optical spectrum based on Eq. (10). Because of the forbidden nature of the optical transition, the equilibrium BSE results in exactly zero optical dipole moment for the lowest energy transition in agreement with the previous HSE06 DFT result [62]. However, it becomes visible by applying non-equilibrium BSE method ($I_{0}^{\text{BSE}}$) at room temperature. The non-equilibrium BSE spectrum appears via $f_{\lambda }^{<}$ in $I_{0}^{\text{BSE}}$ through the thermalisation of electrons and holes. The pseudo-equilibrium occupations equal to

(12) $$f_{nk}=\frac{1}{{\text{e}}^{\frac{\epsilon_{nk}-\mu_{\text{el}}}{k_{\text{B}}T}}+1}\text{,}\;{\bar{f}}_{nk}=\frac{1}{{\text{e}}^{-\frac{\epsilon_{nk}-\mu_{\text{ho}}}{k_{\text{B}}T}}+1}$$

for electrons and holes, respectively. Here *ϵ*_*n* **k**_ is the quasiparticle energy of the state {*nk*} and *μ*_el_ (*μ*_ho_) the chemical potential for electrons (holes). The chemical potential was set for the electrons in such a way that a whole electron is promoted to the excited state manifold as usual in ΔSCF procedure which guarantees a neutral excitation. The chemical potential of the holes is tuned in such a way that the number of holes coincides with that of the electrons excited to the empty states. The non-equilibrium (NEQ) occupations induce a renormalization of the quasi-particle energy levels. It is calculated as

(13) $$\epsilon_{\text{NEQ}}=\epsilon_{\text{EQ}}^{{\text{G}}_{0}{\text{W}}_{0}}+\left(\epsilon_{\text{NEQ}}^{\text{COHSEX}}-\epsilon_{\text{EQ}}^{\text{COHSEX}}\right)\text{,}$$

where the first term is the quasi-particle energy determined at the G_0_W_0_ level of theory using the equilibrium occupations, while the second and the third terms represent the quasi-particle energy at the COHSEX level of theory calculated using the non-equilibrium and equilibrium occupations, respectively.

The calculated *I*^BSE^(*ω*, *T*) spectrum indicates that the phonons associated with the in-plane Jahn–Teller distortion has a very small optical dipole transition moments but the out-of-plane phonons significantly amplify the optical dipole transition moments [86]. As a consequence, the shape of the PL spectrum is governed by the out-of-plane phonons and not the in-plane phonons. We note that the combination of the ^3^*E*′′ electronic state with $A_{2}^{\prime \prime }$ and *E*′′ phonons results in ${\tilde{E}}^{\prime }$ polaronic state which has allowed optical transition towards $A_{2}^{\prime }$ ground state for each polarization of the emitted light. The overall width and shape of the calculated fluorescence spectrum agreed well with the experimental spectrum (see Ref. [86] and references therein) but the calculated spectrum is shifted to lower energy (of about 150 meV). The origin of the discrepancy was not explained in Ref. [86] whether it comes from the G_0_W_0_ approach or the neglect of polaron shift. Nevertheless, the methodology demonstrated its strength in analysing the Herzberg–Teller type optical transitions which is also dominant in the fluorescence spectrum of the singlet states in diamond NV centre [29].

### 3.2 Photo-ionisation thresholds: many-body and temperature effects

Photo-ionisation threshold energies and cross-sections are key properties for such quantum defects for which the qubits are initialised and read out optically. Photo-ionisation may promote an electron from the filled in-gap state to the conduction band (positive ionisation) or an electron from the valence band to the empty in-gap state (negative ionisation). For a given defect in semiconductors and insulators, both process may occur depending on the illumination wavelength. Similar to the neutral photo-excitation processes, phonons may assist the photo-ionisation processes with yielding temperature-dependent photo-ionisation thresholds. *Ab initio* simulations using the usual Born-Oppenheimer approximation with separating the problem of motion of ions and electrons will result in the low-temperature photo-ionisation thresholds that may be not accurate at elevated temperatures.

Photo-ionisation may occur by simply single photon absorption which basially goes the same way as neutral photo-excitation just the initial |Ψ_*i*_⟩ or the final state |Ψ_*f*_⟩ is the host band in the photo-ionisation process, i.e., $\left\langle \Psi_{i}|e\hat{r}|\Psi_{f}\right\rangle$ which is a one-body operator. Another possibility is the Auger-process for such defects that have multiple levels occupied by electrons such as the diamond NV centre (see Figure 1). In semiconductors, the Auger-process is considered to be important for the cases when the number of carriers is high in the band edges of the host material. However, in wide band gap materials, the Auger-process can well compete and be even dominating one when compared to other non-radiative processes such as the electron-phonon coupling because the large energy spacing between the defect level and the band edges leads to too slow multi-phonon processes. The critical matrix element is $\left\langle \phi_{i}\phi_{j}|{\hat{v}}^{\text{eff}}|\phi_{k}\phi_{l}\right\rangle$ where ${\hat{v}}^{\text{eff}}$ is the screened Coulomb interaction appeared also in Eq. (2) where the many-body Ψ wavefunctions are now expressed by the appropriate single-particle wavefunctions *ϕ*s (see Ref. [30] and references therein). The Auger-process is described by this two-body operator.

Another important consideration about describing the photo-ionisation is to distinguish the observable many-body picture and the auxiliary single-particle picture or band structure (see Figure 1). The band structure diagramme is an effective single particle picture including the localised defect states inside the band gap. In other words, band structure virtually plots the single-particle levels of which single-particle wavefunctions build up the many-body Slater-determinant solution. In the case of shallow donor state such as phosphorus donor (P-donor) in Si, the defect level is occupied by a single electron in the band gap, and that electron has a relatively weak exchange-correlation interaction towards the valence band states. Thus, the single electron orbital represents well the many-body total energy with respect to the total energy of the system when the electron is promoted to a higher energy effective mass state. As a consequence, the occupied donor defect level with respect to the conduction band minimum (CBM) can be considered as the photo-ionisation energy (when the relaxation of the ions upon photo-ionisation is neglected or minor). However, if the donor level is occupied by two electrons, e.g., sulfur donor in Si, then the two electrons have considerable Coloumb interaction with each other. As a consequence, once an electron is removed from the doubly occupied donor level then we break the Coulomb repulsion between the two electrons and their contribution to the total energy of the system before ionisation which is missing for the single electron left in the donor level after ionisation. Therefore, the difference betwen the doubly occupied donor level and singly occupied donor level does not correspond to the many-body total energy difference of the neutral and singly ionised sulfur defects. For deep defects with multiple electrons localised on the defect this effect is severe: it results in a strong Coulomb and exchange interactions among the localised electrons. Therefore, the many-body ^3^*A*_2_ level, of which state is Ψ_GS_ Slater-determinant for the diamond NV centre, should not be drawn to the band structure of perfect diamond as ^3^*A*_2_ state already contains all the diamond bands. The false level diagramme with mixing the single particle and the many-body pictures will lead to a false impression about the ionisation energies. For instance, if the ^3^*A*_2_ level is drawn at the position of the ionisation energy (about 2.65 eV at room temperature [87, 88]) w.r.t. CBM then the ^3^*E* level is usually drawn above the ^3^*A*_2_ level with the ZPL energy at 1.945 eV. According to this false consideration, the ionisation energy of ^3^*E* level w.r.t. CBM would be that of the ^3^*A*_2_ minus the ZPL energy which yields about 0.7 eV. The problem with this consideration is that it completely neglects the exchange-correlation effects between the electrons or other words the many-body effects as will be explained below.

**Figure 2:**
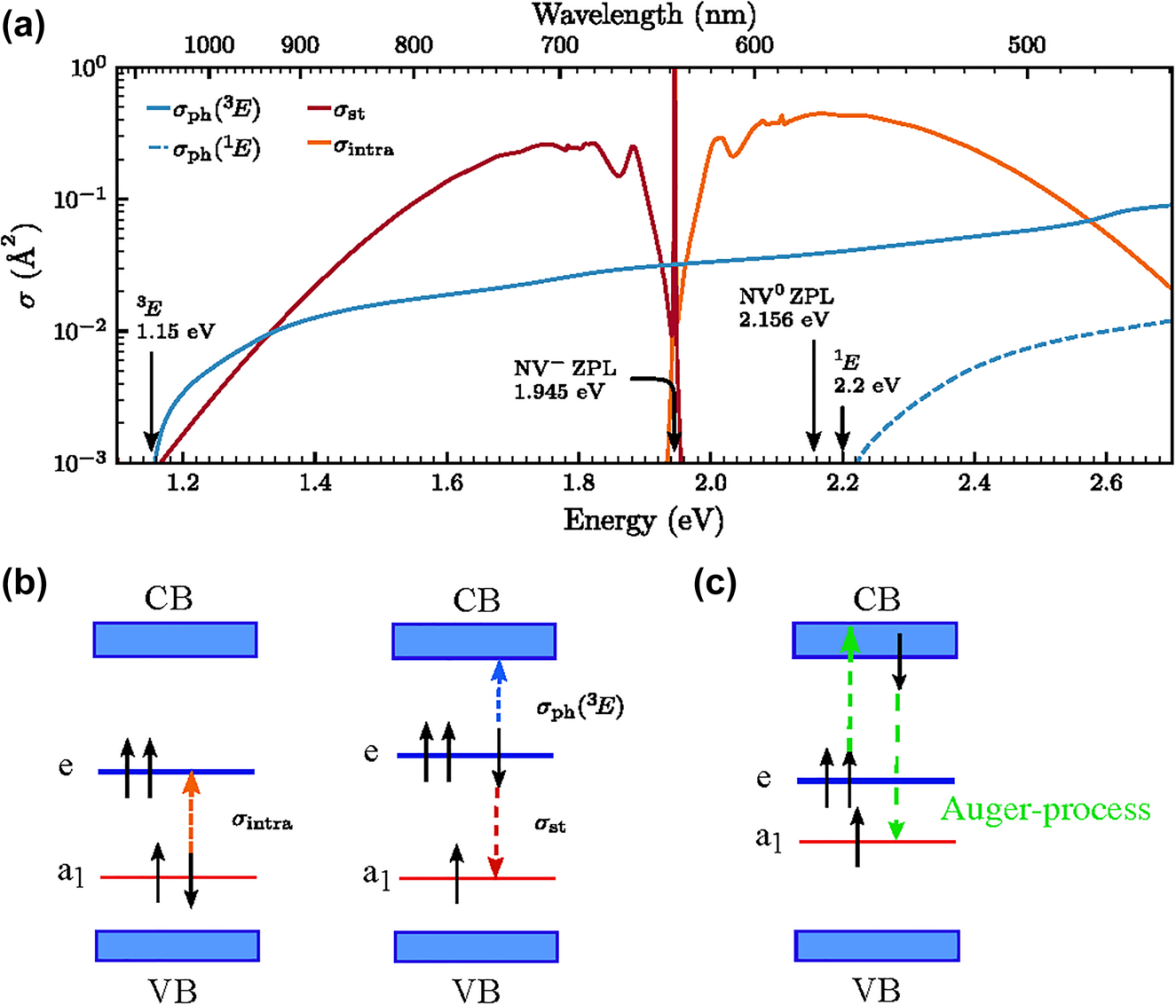
Photo-excitation and ionisation of diamond NV centre. (a) Calculated cross section as a function of photon energy [89]. Solid blue: photo-ionisation from the excited state ^3^*E*, *σ*_ph_; dark red: stimulated emission, *σ*_st_; orange: intradefect absorption, *σ*_intra_; dashed blue: photo-ionisation from the singlet state ^1^*E*. Photo-ionisation thresholds from ^3^*E* and ^1^*E* are indicated (estimated error bar 0.1 eV), together with the experimental values of the ZPL energy for NV(−) and NV(0). (b) Kohn–Sham (single particle) states of the NV(−). The dashed arrows show the excitation processes from the ^3^*A*_2_ ground state (left panel) and ^3^*E* excited state (right panel), where both the stimulated emission and photo-ionisation processes are depicted for the latter. ^4^*A*_2_ shelving state of NV(0) is left after completing the ionisation whereas stimulated emission brings the system back to ^3^*A*_2_ ground state of NV(−). (c) Photo-ionisation via Auger-process after the second photon was absorped. ^2^*E* ground state of NV(0) is left after completing the ionisation.

In the example of diamond NV(−) → NV(0) ionisation process, if we start from the ground state ^3^*A*_2_ electronic configuration of NV(−) (Figure 2(b)) then a single electron from the *e* level is promoted to the CBM which results in the ^2^*E* ground state of NV(0). Starting the ionisation from ^3^*E* state of NV(−), we first have to consider the ^3^*E* state which can be well described as a hole is left in the *a*_1_ level and the double degenerate *e* level is occupied by three electrons (see left panel in Figure 2(b)). Two ionisation processes are viable at this point: (i) direct process with an electron promoted from the *e* level to the conduction band which may leave the defect [89]; (ii) Auger-process occurs after absorption of the second photon where the promoted electron in the conduction band recombines with the *a*_1_ hole, and the energy gain of this process is used to simultaneously promote another electron from the *e* level to a high energy state in the conduction band which finalizes the ionisation process (see Refs. [90, 91] and Figure 2(c)). According to the Slater–Condon rules [92, 93], only one spin orbital can change in the many-body wavefunction upon direct ionisation process described by one-body operator, therefore ^3^*E* of NV(−) arrives at the metastable ^4^*A*_2_ of NV(0) plus an electron in the conduction band by photo-excitation. Since the shelving ^4^*A*_2_ level lies above the ground state ^2^*E* level of NV(0) the ionisation threshold energy of this process starting from ^3^*E* of NV(−) will be higher by about 0.48 eV according to HSE06 calculations [89] then the ionisation threshold energy starting from the ^3^*A*_2_ ground state level minus the ZPL energy of the ^3^*E* ↔ ^3^*A*_2_ optical transition, which finally yields about 1.2 eV ionisation threshold energy [89]. In the alternative Auger-process, the two-body operator nature of the process makes it possible to arrive at the ^2^*E* ground state of NV(0) from ^3^*E* NV(−). If the electron is excited to high energy above the CBM then phonons can very quickly (within picoseconds) cool it down to the CBM which is might be bound by the weakly attractive potential of NV(0) towards the CBM electrons which makes this process viable with about 1 nanosecond rate [90]. In the two-photon absorption process of NV(−), the typical excitation energy is about 4.66 eV (green light at 532-nm wavelength) which subsequently goes through the ^3^*E* level with phonon cooling upon absorption of the first photon and probably a phonon cooling process after absorbing the second photon as explained above. From energetics point of view, it is feasible to arrive at ^2^*E* ground state of NV(0) from the ^3^*A*_2_ ground state of NV(−). A recent experimental study concludes that the two-photon absorption based charge conversion of NV(−) → NV(0) can be well explained by a dominating Auger-process. In the rate modelling, a single-photon optical transition should also occur from the shelving ^1^*E* state to a higher lying state [91]. A strong and broad transition around 2.58 eV from ^1^*E* to ^1^*E*′ has been observed in numerical simulations providing a possible candidate for such a mechanism [41]. ^1^*E*′ can decay to the triplet excited states via an inter-system crossing [30, 94].

Nevertheless, such spin-to-charge-conversion (SCC) protocols exist for diamond NV centre which apply low-power excitation to avoid two-photon absorption processes but dual wavelengths of excitation in which the green illumination is used for the optical cycle between ^3^*A*_2_ and ^3^*E* states whereas longer-wavelength-than-ZPL illumination is applied to ionise it from the ^3^*E* level towards the conduction band [95, 96]. In this case, direct ionisation may occur from the ^3^*E* level. In the seminal work of Razinkovas and co-workers [89], the absolute ionisation cross section from the ^3^*E* state together with the induced emission was calculated for NV centre as a function of the excitation energy (Figure 2(a)). The calculated ratio of the photo-ionisation cross section and the cross section for stimulated emission is greater than 2 for the energy around 1.2 eV and 1.93 eV photo-excitation energies, which agree well with the applied photo-ionisation energies in the SCC experiments [95, 96].

We note that understanding the reionisation process, NV(0) → NV(−), is critical for stabilising the diamond NV(−) qubit state. Typically, the reionisation automatically occurs by the applied green illumination to drive NV(−) by two-photon absorption process which is also subsequential process going through the ^2^*A*_2_ excited state: the first photon absorption brings ^2^*E* to ^2^*A*_2_ and the second photon is then absorbed in the ^2^*A*_2_ excited state. The ^2^*A*_2_ state consists of a hole left in the *a*_1_ level and two electrons in the *e* level. The photo-excitation of the ^2^*A*_2_ excited state of NV(0) may also occur by either direct process (promoting an electron from the valence band to the empty *a*_1_ defect level in the gap) or Auger-process (occupying the in-gap *a*_1_ hole by an electron from the in-gap *e* level and then promoting an electron from the valence band to the empty *e* defect level in the gap). Both processes leave a hole in the valence band. In the direct process, the system arrives at the ground state of NV(−) because of the alluded Slater–Condon principle [91]. On the other hand, the Auger-process enables to arrive at the ^1^*A*_1_ and ^1^*E* singlet states of NV(−) too, beside the ^3^*A*_2_ ground state. The energy cost of these processes varies with the final state. The calculated adiabatic acceptor charge transition level of the NV defect is at about 2.75 eV from the conduction band edge [97, 98], whereas the calculated energy gap between the ^3^*A*_2_ ground state and ^1^*A*_1_ state is at about 1.6 eV (see Ref. [30] and references therein). The total energy cost to convert the NV(0) ground state to the ^1^*A*_1_ NV(−) excited state is then about 4.3 eV which coincides with twice the ZPL energy of NV(0) [91]. This means that a special excited state of ^1^*A*_1_ of NV(−) binding a hole resonant with the valence band maximum (VBM) develops. This hole is Coulombically bound which is a special bound exciton state or Rydberg state which has been observed for the SiV defect [11] and has been recently implied and modelled for the ^3^*A*_2_ plus bound hole system for the NV(−) defect [99, 100]. The bound hole is weakly localised with following the effective mass theory. By even taking into account the possible relaxation energy of the ions caused by the change in the electronic states, we may claim that a green laser excitation can reach the ^1^*A*_1_ plus bound hole state of NV(−) by two-photon excitation of NV(0). Scattering rather to ^1^*E* and ^3^*A*_2_ states of NV(−) via Auger-process leaves a hole deep in the valence band at around 1.2 eV and 1.6 eV from the VBM, respectively. According to the calculations [91], a resonant *a*_1_ state broadened by the diamond bands occurs in this energy region which is originated from the dangling bond orbitals of the carbon and nitrogen atoms near the vacant site. The resonant state is weakly localized unlike the usual diamond bands that are completely delocalized. This should lead to a larger direct and Auger-ionization rates of NV(0) than those of NV(−) because no such a high-energy resonant state sharing the same spin state with that of the ground state exists in the conduction band, critical in the photo-ionisation of NV(−). Interestingly, the Auger-process should lead to a preferential occupation of the *m*_*s*_ = 0 state via spin-selective intersystem crossings between the ^1^*E* state and the ^3^*A*_2_ spin states; however, direct ionisation would result in 1/3:2/3 relative population of the *m*_*s*_ = 0:*m*_*s*_ = ±1 states of ^3^*A*_2_ [91]. The *ab initio* calculation of the rates of the direct and Auger-processes requires an accurate computation of the excited states of NV(0) which has not yet been solved as we briefly discussed it in Section 3.1.

The afore-mentioned *ab initio* calculations are based on the global energy minimum of the APES in the appropriate electronic structures and charge states which correspond to the zero kelvin solution. However, the photo-ionisation tresholds of diamond NV centre are often observed at room temperature. Principally, the effective ionisation threshold energies may change as a function of temperature. A very characteristic example is the silicon carbide (SiC) divacancy defects. In particular, four divacancy configurations with similar electronic structures occur in the so-called 4H polytype of SiC [101–103], some of them well observable at room temperature [104, 105]. We note that 4H SiC exhibits a band gap of about 3.3 eV that can host visible and near-infrared colour centres acting as qubits [11, 106, 107]. The neutral divacancy defects in 4H SiC have isovalent electronic structure to diamond NV centre as depicted in Figure 1 but the energy gaps between the levels are about twice as smaller, thus they produce near-inrared emission. It was found that upon photo-excitation of the defect it falls to a “dark” shelving state due to two-photon absorption or other complex processes. This shelving state has been finally identified as the negative charge state of the defect [108–110]. Close to cryogenic temperatures, a shorter wavelength laser beam was applied to drive the divacancy back to the neutral qubit state by promoting an electron from the *e* level to the CBM [110]. It was later found that at elevated temperature the quenching of the fluorescence of 4H SiC divacancy (V2) defects does not occur and they remain optically stable [111]. This can be interpreted as the photo-ionisation threshold energy associated with the V2(−) → V2(0) process is decreasing upon raising the temperature.

The modelling of temperature-dependent photo-ionisation processes requires the following considerations: (i) the CBM and VBM of the host material may shift with temperature, (ii) the formation enthalpy so the charge transition level of the defect with respect to the band edges may shift with temperature via the vibration entropy, and (iii) the phonon assisted ionisation process may be activated by raising the temperature. The first effect is basically the temperature-dependent electron-phonon renormalization of the bands. This can be computed by the many-body perturbation theory on the electron-phonon coupling [112–114] that was applied to 4H SiC [115]. The usual case is that the CBM and VBM shifts down and up with raising the temperature, respectively, leading to an effective decrease of the fundamental band gap. It was found by *ab initio* calculations that the CBM of 4H SiC shifts down by about 5 meV from zero kelvin to room temperature [115]. In other materials with low Debye-temperature, this effect could be significantly enhanced. The second effect assumes an *ab initio* treatment of the thermodynamic properties of solid-state defects that was thouroughly discussed in Ref. [34]. Here, the key effect is the vibration entropy correction to the formation energy of the defect,

(14) $$F^{q}\left(T\right)=\sum_{i}\left\{\frac{1}{2}\hbar \omega_{i}+k_{\text{B}}T\ln\left[1-\exp\left(-\frac{\hbar \omega_{i}}{k_{\text{B}}T}\right)\right]\right\},$$

where *ℏ* and *k*_B_ are the reduced Planck constant and Boltzmann constant, respectively, and *ω*_*i*_ is the frequency of the *i*th phonon mode in charge state *q* of the defect at the given *T* temperature. The first term in Eq. (14) is the zero-point energy. The actual values of *F*^*q*^(*T*) could differ for a given defect in various charge states (Δ*F*(*T*) = *F*^−^(*T*) − *F*^0^(*T*)) which results in a shift of charge transition level, here with respect to CBM, $\left(E_{-/0}^{\text{CBM}}\right)$. This correction may increase or decrease the effective photo-ionisation threshold. The total correction is then,

(15) $$E_{-/0}^{\text{CBM}}\left(\text{corr}\right)=E_{-/0}^{\text{CBM}}-\Delta E^{\text{CBM}}\left(T\right)+\Delta F\left(T\right)\text{.}$$

The value of $E_{-/0}^{\text{CBM}}\left(\text{corr}\right)$ corresponds to the ionisation threshold energy without involvement of phonons in the photo-ionisation process [111]. By raising the temperature the phonon excited states are occupied and may contribute to the ionisation process which may be considered as an phonon-assisted optical transition between the neutral defect binding an electron in the CBM and the negatively charged defect where we neglect the interaction of the CBM elecron with the rest of the electrons. This effect may strongly contribute to the reduction of the photo-ionisation threshold at elevated temperatures. The effect is analogous with the appearance of the phonon bands at lower energies than ZPL energy in the absorption spectrum of the defect at elevated temperatures. The phonon-assisted optical spectrum can be calculated within Franck–Condon approximation at *ab initio* level for defective supercell models (see Refs. [30, 116] and references therein). The calculated temperature-dependent ionisation threshold energies for the divacancy centres in 4H SiC based on this theory were reported in Ref. [111]. Although, the very long wavelength acoustic phonons were not calculated in that study for the vibration entropy and the Franck–Condon theory that shed doubts on the convergence of the results, nevertheless, the equal or similar contributions of the temperature-dependent vibration entropy and Franck–Condon terms are demonstrated, and it provided explanation about the photo-stability of 4H SiC divacancy qubits at room temperature [111].

### 3.3 Supercell modelling of defects: extrapolation to dilute limits

In the supercell modelling of point defects the goal is to describe isolated point defects. In practice, the size of the supercell is limited up to about 10,000 atoms for KS DFT calculations due to computational resources. By applying accurate hybrid KS DFT functionals the size of the supercell is further reduced to about 1000 atoms. This size of the supercell suffices to obtain accurate results for deep defects because the defect induced wavefunctions and strain fields decay relatively fast from the core of the defect. However, shallow states with weakly localised character of defect wavefunctions such as the case of P-donor in Si, require extrapolation to dilute limits. For deep defects, certain properties also call for special treatments in the supercell formalism that we non-exhaustively list here: charge correction for charged defective supercells, acoustic phonon couplings to electron orbitals and spins, excitation and ionisation towards electronic bands of the host.

#### 3.3.1 Charge correction schemes: a recent breakthrough

In our previous review paper [30], the charge correction for charged defective supercells was thouroughly discussed. To sketch here the problem and the possible solution we note that the introduced defect charge is neutralized by a compensating jellium charge in the supercell. The charge of the defect and the jellium background charge interact with their periodic images that goes with the leading point charge Coulomb interaction of which energy scales with *L*^−1^, where *L* is the edge of the simple cubic supercell. This theory already gives a hint about the expected scaling property in the correction of the total energy to the isolated defect or dilute limit. Indeed, the total energy of the charged defective supercells converges notoriously slow with the supercell size as indicated by the simple theory above. By applying charge corrections to the total energy, the convergence can be well accelerated. In the previous years (see Refs. [30, 34, 117] and references therein), many *a posteriori* schemes have been developed for the correction of the total energy of charged defective supercells in 3D and 2D models. In those schemes, the possible change in the character of the wavefunction due to the charge correction was not taken into account that might lead to qualitatively wrong results in notorious cases. This problem is in particular severe for slab models of crystal surfaces with negative electron affinities. Recently, a self-consistent potential charge correction (SCPC) method was developed to heal this issue which goes beyond the previous *a posteriori* total energy corrections of the charged defects, and they derive the KS potential associated with the charge correction and self-consistently solve the constructed KS DFT equations [117].

The SCPC method yield the corrective potential (*V*_cor_) in an iterative manner: (i) the distribution of the extra charge in the supercell (*δρ*) is determined, (ii) the corresponding periodic electrostatic potential (*V*_per_) is calculated, (iii) the potential for the same but isolated charge distribution (*V*_iso_) is determined by using open (Dirichlet) boundary conditions, and finally, (iv) *V*_per_ and *V*_iso_ are used to determine the corrective potential *V*_cor_, which is added to the total electronic potential. It should be noted that SCPC always aligns the final potential, considering the difference between the electrostatic potentials of the charged and the reference system far away from the defect position (Δ*V*).

The method was originally built in the VASP code but an interface has been developed to Quantum Espresso code too. The SCPC method was applied to diamond slab model with NV centre where the (100) diamond surface was terminated by hydrogen. It was shown that without SCPC method the negative charge of the defect artificially pull down the bands of the surface states, so-called image states, which results in a false electronic structure even in large supercells [117]. Here, the self-consistent correction of the potential is essential. We note that the self-consistent correction is not a must for many defects in 3D solids and *a posteriori* charge correction schemes can provide qualitatively good results. Despite the self-consistent nature of the correction, the SCPC method does show supercell size dependence which comes from the fact that the character of the underlying (localized) defect wavefunctions may change with supercell size due to the defect-induced strain fields and other factors, so supercell size scaling is still necessary with this method but converges faster than the *a posteriori* total energy correction schemes [117].

#### 3.3.2 Embedding of long wavelength phonons in the finite size of the supercell

The long-wavelength acoustic phonons could contribute to the phonon sideband of the optical spectra of defects. Audrius Alkauskas and co-workers developed an embedding method to include the electron-phonon coupling in the optical transition [116] that they applied to the optical transition between the triplet states of diamond NV centre [79, 116] which has been also implemented in the sf-TDDFT study for the absorption spectrum of the singlet states [78]. Similar treatment is advisable for the temperature-dependent photo-ionisation spectrum [111].

#### 3.3.3 Reconstruction of the deep-energy valence and high-energy conduction bands

Accurate absorption cross section calculation of excitation from/to deep levels to/from solid-state bands within supercell formalism requires special attention due to band folding in the Brillouin-zone [118]. This was recognized by Razinkovas and co-workers when they studied the absorption cross section for the photo-ionisation of diamond NV centre [89]: they found minigaps in the conduction band which affected the calculated absorption cross section at the given high energy excitation. They used the following technique to circumvent this problem: (i) they identified the folded band in the Brillouin-zone of the primitive diamond cell as described in Ref. [118], (ii) then they averaged out the calculated absorption cross section values closest to the corresponding *k*-space within the energy region of about 0.08 eV. The resulted photo-ionisation absorption spectrum is then converged well.

#### 3.3.4 Treatment of spatially extended defect wavefunctions: beyond effective mass theory

KS DFT calculation of the properties of shallow donors in Si is computationally very challenging because of the spatial extension of the donor wavefunction. Accurate calculation requires hybrid HSE06 functional, e.g., Ref. [119], which becomes extremely difficult to carry out for sufficiently large supercell sizes. As was done for the total energy correction of charged defective supercells (see Refs. [30, 34, 117] and references therein), one can apply a strategy by studying theoretically and numerically the scaling of the given property as a function of the supercell size, and then extrapolate the result to the dilute (isolated defect) limit. In many cases, the exact scaling properties are unknown or they are too much complex because different effects (electron density and charge distribution, strain field distribution) are intertwinned and they often depend on the local electron density distribution of the defect that might change by increasing the size of the supercell.

In practice, numerical KS DFT investigations could lead to converged results which requires sufficiently large number of sampling points for defining the scaling law, i.e., ideally up to supercell size with about 10,000 atoms. This is prohibited by the accurate HSE06 functional so a typical strategy is to calculate the scaling by the affordable PBE functional and for a limited range of supercell sizes to compare the scaling with HSE06 and PBE functionals to verify the scaling function.

Swift and co-workers studied the shallow group-V dopants of Si by supercell KS DFT calculations [119]. These dopant introduces a singly occupied electron the state split from the CBM. In semiconductor physics, these states are described by the so-called effective mass theory from Kohn and Luttinger which treats the dopant potential as a positively charged Coulomb potential which binds an electron of which state can be described the linear combination of the CBM valleys. The solution of this system results in a Rydberg or hydrogenic series of excitation energies until it converges to the ionisation level. The ionisation or binding energy of the electron (*E*_*b*_) can be defined as the lowest energy level *ϵ*^donor^ (1*s* like envelop function) with respect to CBM of the perfect crystal (*ϵ*^CBM^). The validity of this approximation from many-body electron-phonon point of view was briefly discussed in Section 3.2. It is known in experiments (see Ref. [119] and references therein) that the ionisation energies of various group-V dopants in Si differ. Therefore, a so-called “central cell correction” was introduced to the effective mass theory which assumes that the 1*s* ground state wavefunction has the largest overlap with the dopant ion’s attractive potential which will pull down its energy level with respect to the purely hydrogenic solution (enlarging the donor ionisation energy). As the potential of the dopant ion is characteristic to the dopant within short range around the dopant ion, thus the “central cell correction” will be dopant dependent. The central cell correction is merely semiempirical correction to the effective mass theory of shallow donors and acceptors, where the acceptor levels are measured with respect to VBM. In KS DFT calculations, the ionisation energies can be calculated at *ab initio* level free from any assumptions on the nature of the potential induced by the dopants. It can be estimated from the effective mass theory that the 1*s* donor wavefunction in Si will decay at around 55 Å from the position of the dopant which would require a supercell of about 64,000 atoms to accommodate the donor wavefunction without significant overlap. This definitely calls to apply a scaling procedure even for PBE DFT functional.

Swift and co-workers [119] applied scaling method for calculating *E*_*b*_ of arsenic (As) and bismuth (Bi) donors in Si by PBE and HSE06 functionals. The calculations were carried for supercells from 64 to 2744 atoms at PBE level and for supercells from 64 to 1000 atoms at HSE06 level. They calculated *E*_*b*_ at a given size of the supercell as

(16) $$E_{b}=\epsilon^{\text{CBM}}-\epsilon^{\text{donor}}+e\Delta V\text{,}$$

where Δ*V* potential alignment between the defective and perfect supercells appears the charge correction of defects in Section 3.3.1. Interestingly, the Δ*V* does not show a clear monotonous decay with increasing size of the supercell which was not explained [119]. The scaling of the HSE06 data was carried out as

(17) $$b_{\text{HSE}06}=b_{\text{PBE}}^{\text{fit}}-\frac{1}{2}b_{\text{PBE},\delta^{\text{ex}}}^{\text{fit}}+\frac{1}{2}b_{\text{HSE}06,\delta^{\text{ex}}}^{\text{fit}}\text{,}$$

where $b_{\text{PBE}}^{\text{fit}}$ is the slope fit to the PBE binding energies, $b_{\text{PBE},\delta^{\text{ex}}}^{\text{fit}}$ is the slope fit to the PBE exchange splitting, and $b_{\text{HSE}06,\delta^{\text{ex}}}^{\text{fit}}$ is the slope fit to the HSE06 exchange splitting. The exchange splitting was defined as the difference between the spin-up and spin-down eigenvalues of the donor state. Subtracting half of the exchange splitting from the binding energy yields a spin-averaged value which is corrected between the PBE and HSE06 results with assuming that the supercell-size dependence obtained in PBE for the spin-averaged case applies also to the HSE06 values. They obtained 54 meV and 67 meV binding energies for As and Bi donors, respectively, in very good agreement with the experimental data at 53.9 meV and 70.9 meV, respectively [119].

The neutral donor defects introduce *S* = 1/2 spin that can interact with the nuclear spins of the dopant or the proximate ^29^Si *I* = 1/2 nuclear spins which is called hyperfine interaction. Actually, the interplay between the electron and nuclear spins could represent qubit states in these systems, therefore, understanding this interaction as a function of electric field and strain is highly important (see Ref. [120] and references therein). This hyperfine interaction can be generally written as

(18) $$H_{\text{hyp}}=\hat{S}A\hat{I}\text{,}$$

where *A* is the hyperfine tensor and $\hat{S}$, $\hat{I}$ are the electron spin, nuclear spin vector operators, respectively. The Fermi-contact and the dipole-dipole terms of the hyperfine tensor can be respectively written as

(19) $$\begin{array}{ll} A_{ab}^{\left(n\right)} & =\frac{2\mu_{0}}{3}g_{e}\mu_{\text{B}}g_{n}\mu_{n}\frac{n_{s}\;\left(R\right)}{S}+\frac{\mu_{0}}{4\pi }g_{e}\mu_{\text{B}}g_{n}\mu_{n}\frac{1}{S}\\ & \;\times \int \frac{3r_{a}r_{b}-r^{2}\delta_{ab}}{r^{5}}n_{s}\;\left(r\right)d^{3}r\text{,} \end{array}$$

where $n_{s}\;\left(r\right)$ is the electron spin density, **r** is the vector between the electron spin and nuclear spin at **R**, *g*_n_ is the nuclear *g*-factor, and *μ*_n_ is the nuclear magneton for a given nucleus *n*. For the ground state 1*s* donor wavefunction, the Fermi-contact term predominates which depends on the localization of the donor wavefunction at place of the dopant. Swift and co-workers found that (i) PBE produces too low hyperfine constants for shallow donors in Si, (ii) reliable values are obtain for supercell size of 512 atoms or larger number of atoms, and (iii) in that size range the hyperfine constant for the dopant atom scales as *L*^−1^. The final HSE06 values are 132.5 MHz and 1262 MHz for As and Bi spins, respectively, compared to the experimental data at 198.3 MHz and 1475 MHz, respectively [119]. They also found that the electric field gradient, *V*_*zz*_, around the dopant atom, so the quadrupole interaction strength *C*_*Q*_ = 3*eQ*_atom_*V*_*zz*_/4*h* is vanishingly small (1 MHz), where *h* and *e* are the Planck-constant and the charge of the electron, and *Q*_atom_ is the nuclear electric quadrupole moment of the dopant atom. Furthermore, they also studied the strain dependence of the contact hyperfine tensor of the Bi dopant, which generally reads [120] as

(20) $$\begin{array}{ll} A/A_{0} & =1+\frac{K}{3}\left(\varepsilon_{xx}+\varepsilon_{yy}+\varepsilon_{zz}\right)+\frac{L}{2}\left[{\left(\varepsilon_{yy}-\varepsilon_{zz}\right)}^{2}\right.\\ & \;\left.+{\left(\varepsilon_{xx}-\varepsilon_{zz}\right)}^{2}+{\left(\varepsilon_{xx}-\varepsilon_{yy}\right)}^{2}\right]\\ & \;+N\left(\varepsilon_{yz}^{2}+\varepsilon_{xz}^{2}+\varepsilon_{xy}^{2}\right)\text{,} \end{array}$$

where *A*_0_ is the value in the absence of strain, *K* coupling is responsible for the hydrostatic strain, *L* coupling and *N* coupling describes the uniaxial and shear strain effects, respectively. Swift and co-workers only focused to the hydrostatic strain in their *ab initio* study: they found that *K* scales the same in HSE06 and PBE functionals in relatively small supercells, thus the error in PBE in the difference of absolute values of hyperfine constants as a function of strain is cancelled. As a consequence, the PBE scaling can be applied for larger supercell for extrapolation to the dilute limit. They obtained *K* = 20.2 which is close to the experimental data at *K* = 19.1 (see Refs. [119, 120]).

**Figure 3:**
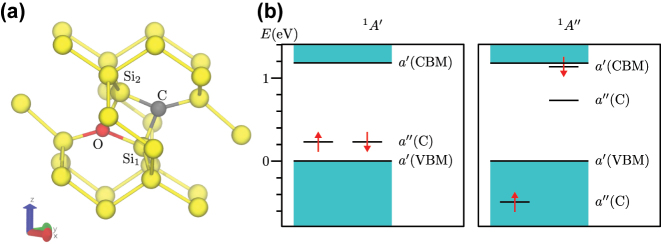
C-centre in silicon. (a) The atomic structure of the C_i_O_i_ defect complex, consisting of neighbouring carbon (C_i_ − Si_1_) and oxygen (O_i_ − Si_2_) split-interstitial defects associated with C-centre in Si. (b) Kohn–Sham level structure of the C_i_O_i_(0) defect ground state (^1^*A*′) and singlet (^1^*A*′′) excited state in the spin-polarised HSE06 calculation. VBM, CBM and C label the valence band maximum, the conduction band minimum, and the dangling bond orbital of the carbon atom, respectively.

In the afore-mentioned examples, the defect wavefunction is spatially extended in the electronic ground state. Perhaps, it is not a common knowledge among scientists coming from the quantum optics field that similarly extended wavefunctions could exist in the electronic excited states. Again, the best example is the most studied small band gap material, silicon. As silicon has a band gap of 1.215 eV at cryogenic temperatures, there is a little room to introduce multiple levels by deep defects. Defects may introduce only a single occupied deep level in the fundamental band gap of Si where the electron could be promoted from the in-gap defect level to CBM. In this case, the defect can be described as a positively charged centre which Coulombically binds an electron split from CBM. This definitely shows a similarity to the shallow donor states in Si. For example, the photoluminescence C-centre in Si shows a sharp ZPL at 789 meV for which photoluminescence excitation (PLE) measurements revealed a hydrogenic or Rydberg series of excited states [121]. Later it was shown that this type of PLE features is common for other deep optical centres of Si that was called “pseudo-donor” model [122, 123]. Recently, the pseudo-donor model of C-centre in Si has been confirmed by HSE06 calculations [124]. The neutral C_*i*_O_*i*_ defect associated with this optical centre indeed produces a deep level in the fundamental band gap (see Figure 3), and the calculated ZPL energy at 750 meV by ΔSCF method agreed well with the experiment. In this calculation, 512-atom supercell was employed with the same correction in the total energy of the excited state of the defect (56 meV) as for the positively charged defect. The reasoning behind this method was the following. The excited state involves a spatially extended wavefunction. The scaling property of the total energy of the excited state was assumed to go similarly to that of the positively charged defect within the accuracy of about 50 meV because the extended electron occupying the state split from CBM could behave similarly to all the crystalline valence bands of the system which leaves a positively charged core; in other words, the pseudo-donor electron does not “shield” the positively charged core. There is a further note here about the accuracy of ΔSCF method. In a 216-atom supercell, the results from ΔSCF method and GW + BSE method were compared. It was found that GW + BSE confirmed the composition of the exciton as the deep hole state and an electon state split from CBM and the vertical excitation energies were within 11 meV. Thus, ΔSCF method can be applied for the bound exciton excitation too which is important to calculate the Stokes-shift upon excitation as no quantum mechanical force calculation has been yet implemented to GW + BSE methods. For the case of shallow donors in Si, the geometry change upon ionisation was neglected. However, this cannot be neglected for the fluorescence spectrum of deep defects. Indeed, the sharp features in the phonon sideband of the PL spectrum could be well reproduced by applying the Franck–Condon theory [124]. According to the calculations, C-centre is a potential building block of quantum repeaters in the telecom L-band [124].

**Figure 4:**
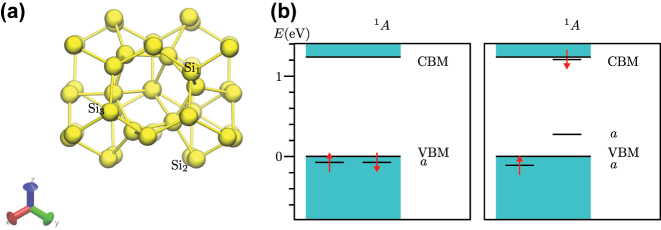
W-centre in silicon. (a) The atomic structure of the tri-interstitial (I_3_) complex associated with W-centre in Si. (b) Kohn–Sham level structure of the I_3_(0) defect ground state (^1^*A*) and (^1^*A*) excited state in the spin-polarised HSE06 calculation. VBM and CBM label the valence band maximum and the conduction band minimum, respectively.

Another deep optical centre in Si, the W-centre, has been recently isolated as a single quantum emitter with ZPL wavelength close to the telecom region at 1218 nm (1.018 eV) (see Ref. [20] and references therein). The defect contains a complex of three self-interstitial silicon atoms. The most stable configuration, so called I_3_-V configuration with *C*_3_ symmetry, has been recently identified by HSE06 calculations where the relative stability changes with respect to PBE calculations [20], which we call here I_3_ for the sake of simplicity. The electronic structure of the neutral I_3_ is very interesting: it shows a single resonant *a* level at 73 meV below VBM (see Figure 4). At first glance, this defect may be considered as electrically and optically inactive. However, after ionisation, the unoccupied defect level emerges inside the band-gap, and the (+/0) charge transition level is at 55 meV above VBM after applying charge correction in the total energy of the positively charged defect. As the stability of the positive charge state is confirmed, the positively charged defect may Coulombically bind an electron with the state split from CBM, alas, the neutral excitation of I_3_ is a bound exciton with a strongly localized hole on the defect and a loosely bound electron [20]. The pseudo-donor nature of the defect was confirmed by HSE06 ΔSCF calculation in 512-atom supercell. The estimation of the ZPL energy was based on the full geometry relaxation of the electronic ground and excited states with scaling of supercell sizes,

(21) $$E_{\text{ZPL}}\left(L\right)=A/L+B/L^{3}+C\text{,}$$

where *L* is the side of the simple cubic supercell, *A*, *B*, *C* are fitting constants, where *C* value corresponds to the dilute limit. *L* was varied between the supercells of 216-atom and 8000-atom for PBE calculations and up to 1000-atom for HSE06 calculations. It was found that the 216-atom supercell results do not fit to the trend and should be ignored. The idea of the formula in Eq. (21) is that the excited state requires charge correction. Since too few data points could be calculated at HSE06 level, it was assumed that the PBE results well reproduce the electrostatics of the problem, and the *A* and *B* fit results can be used for HSE06 data points. This procedure finally yields *C* = 1.102 ± 0.003 eV which is within 0.1 eV when compared to experimental data.

The pseudo-donor or bound exciton excitation can occur in wide band gap semiconductors too. A very nice example is the so-called *D*_*I*_ centre in 4H SiC [125, 126]. The optical activity of the defect is identified as the silicon antisite [127, 128] which is an isovalent centre with producing a deep donor level in the fundemental band gap. The defect can be positively charged and then it can Coulombically bind an electron from CBM with producing Rydberg series of excited states [125]. It can be expected that similar bound exciton excited states may be found in diamond.

Indeed, a recent study has identified Rydberg series in the optical spectrum of the diamond neutral silicon-vacancy [SiV(0)] defect in a joint experimental and theoretical study [11]. Interestingly, optical spin-polarisation and ODMR signals could be also observed through their bound exciton states [11] which makes the analysis of these states highly important as this defect can be isolated in diamond as a near-infrared single photon emitter [129].

**Figure 5:**
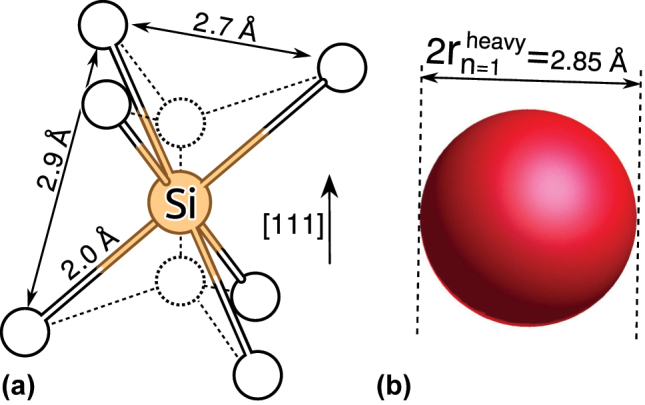
Extension of effective mass state in diamond SiV(0) defect. (a) Geometry of the SiV(0) defect in diamond as optimized by HSE06 in the ground state. The dashed circles represent the missing C atoms, i.e., adjacent vacant sites or V_2_. (b) The Bohr-diameter of the heavy-hole for the *n* = 1 or 1*s* effective mass state. Apparantly, the dangling bond orbitals in V_2_ are confined to the same space as the 1*s* effective mass state.

**Figure 6:**
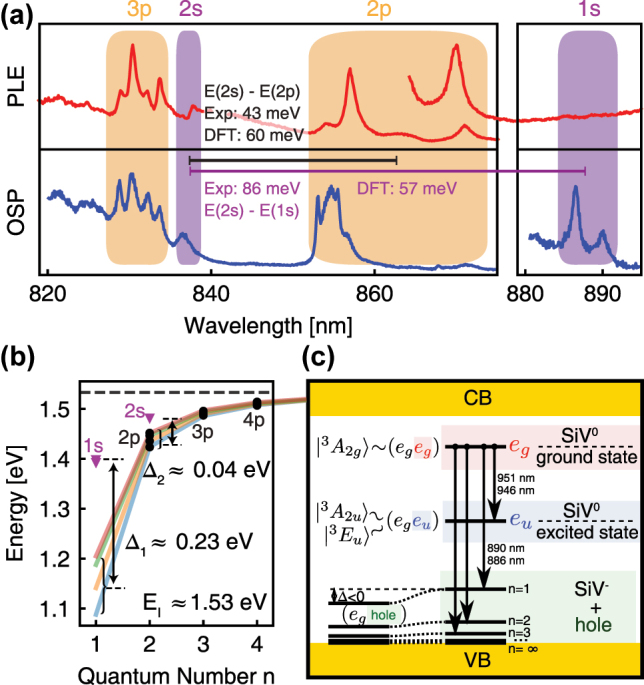
Experimental signals and effective mass theory for the excited state of diamond SiV(0) defect. (a) Experimental photoluminescence excitation (PLE) and optical spin-polarisation (OSP) spectra from Ref. [11]. The OSP can be observed in the spin-polarisation of the ground state towards the *m*_*s*_ = 0 electron spin state in the electron paramagnetic resonance spectrum. (b) Scaling of the peak positions extracted from PLE in (a). The fit uses Rydberg scaling *E*_*n*_ = *E*_*I*_ − *E*_*y*_/*n*^2^ associated with the effective mass states, where *n* refers to the principal quantum number. Due to similar fine structures of 2*p* and 3*p* states, we fit different fine structure transitions (wiggles in the PLE and OSP curves) separately corresponding to the different coloured curves. The fitted ionization energy (*E*_*I*_) and Rydberg energy (*E*_*y*_) are 1.53 eV and 0.4 eV, respectively. The horizontal dashed line indicates the fitted ionization energy. States with “s”-like character are taken from spin-polarisation measurements, and are shown with triangles. Δ_1_ and Δ_2_ are energy deviations for 1*s* and 2*s* states compared to the fitted Rydberg scaling that involve both central cell correction and the localized phonon energy. (c) Proposed bound exciton model for the higher-lying excited states showing orbital ground and excited states and BE states at higher energies in the hole picture. The lower levels closer to the valence band maximum for electrons require higher excitation energy.

The electronic structure of SiV(0) in diamond was already briefly described in Section 3.1.1 that we extend here before we proceed to the discussion of the bound exciton states. In SiV(0) defect, Si atom sits in the inversion centre of diamond so the defect can be rather described as a *V*_2_ defect with six carbon danglings bonds whereas the “dopant” Si ion resides in the empty space of *V*_2_ with the farthest distance from these six carbon atoms (see Figure 5). The six carbon dangling bonds create a double degenerate *e*_*u*_ level resonant with the valence band and a double degenerate in-gap *e*_*g*_ level occupied by two eletrons. This forms the ^3^*A*_2*g*_ ground state. The usual optical activity is associated with promoting an electron from the *e*_*u*_ level to the *e*_*g*_ level for which the optically allowed ^3^*E*_*u*_ → ^3^*A*_2*g*_ transition yields the ZPL energy at 1.311 eV (see Figure 6(c)). This energy is much smaller than the ionisation threshold energy at 1.53 eV which corresponds to the neutral to the negative charge transition (see Ref. [11] and references therein). It is important to notice the selection rules of optical centre with inversion symmetry that the optical transition is only allowed by changing the parity of the participating wavefunctions.

By increasing the excitation energy above the ZPL at 1.311 eV but below the ionisation threshold energy at 1.53 eV, one can excite the hole from the VBM which results in a SiV(−) defect plus a loosely bound hole, i.e., a bound exciton state of SiV(0). Generally, analysing the hole bound exciton spectrum has an increasing complexity over that of electron bound exciton spectrum because of the orbital degeneracy of VBM at the Γ-point which results in an effective spin–orbit interaction. A detailed description is beyond the scope of the present review paper. We rather defer the readers to the supplemental material of Ref. [11] which is now very briefly summarized here. The three-fold degenerate VBM of diamond sligthly splits due to the defect potential resulting in *a*_1*g*_ and *e*_*g*_ bands, where *a*_1*g*_ band lies above *e*_*g*_ band. The VBM splitting also affected by the spin–orbit coupling which has similar energy as the crystal field splitting induced by the defect potential [11]. The spin–orbit coupling creates light-hole, heavy-hole and a split-off hole in the VBM, where the heavy-hole has the shortest Bohr-radius effective mass state orbitals [11]. According to the theory from Thiering and Gali [11], the 1*s*, 2*s*, … effective mass states will transform as *A*_1*g*_, whereas 2*p*, 3*p*, … effective mass states will transform as *A*_2*u*_ and *E*_*u*_, and 3*d*, 4*d*, … effective mass states will transform as *A*_1*g*_ and *E*_*g*_. As a consequence, only the *p*-type effective mass states can be optically excited from the *A*_2*g*_ ground state. The lowest energy 1*s* effective mass state may be observed with the contribution of *u*-type of phonons as phonon-assisted optical transition. Since the translation motion of Si ion in the void of V_2_ transforms with *u* odd-parity [130] the 1*s*, 2*s*, … as well as 3*d*, 4*d*, … effective mass states could be optically excited via the *A*_2*u*_ quasi-local phonon mode of the Si ion which is about 43.4 meV according the PBE calculations [11]. Indeed, the 1*s* effective mass state was not observable in the PLE spectrum but well detectable in the optical spin-polarisation spectrum mediated by the Si-ion vibrations [11] (see Figure 6(a)).

By applying Rydberg scaling to the experimental data (Figure 6(b)), one can find that the expected binding energy of the 1*s* level is about Δ_1_ − 0.04 = 0.19 eV deeper when the experimental data is corrected with the phonon energy of the Si ion vibration.

An important observation is that the central cell correction makes the 1*s* level shallower (i.e., its binding energy becomes smaller) than the value of the effective mass theory, in stark contrast to the case of shallow donor and acceptor dopants in semiconductors. The central cell correction energy can reach hundreds of meV for deep defects in diamond.

**Figure 7:**
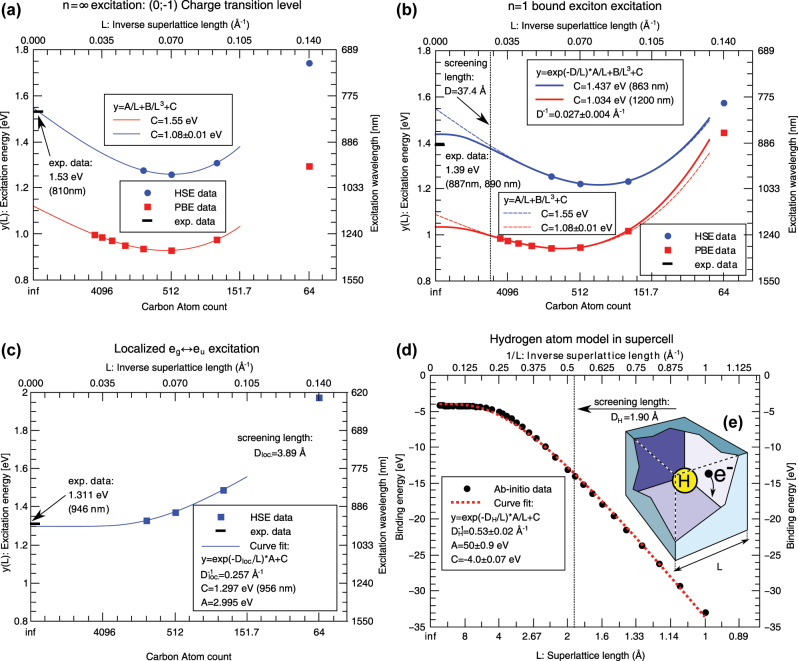
Excitation processes of SiV(0). (a) Charge transition level of SiV between neutral and negative charge states by means of HSE06 and PBE functionals. In the dilute *L* → +∞ limit, the HSE06 results (1.55 eV) agree with the experimental data at 1.53 eV. (b) *n* = 1 1*s* bound exciton excitation by means of HSE06 and PBE functionals. Here we can see that the HSE06 limit at *L* → +∞ with screening included can explain the experimentally data at 1.39 eV. (c) Scaling of the *e*_*g*_ ↔ *e*_*u*_ excitation process by means of HSE06 functional. (d) Total energy of the hydrogen atom in a Hartree–Fock Γ-point calculation in a simple cubic supercell as a function of the size of the supercell. (e) Schematic of the hydrogen atom in vacuum. The electron is effectively closed into a *L*^3^ box. However, it is effectively not a box as it warps around its edges due to the interaction with its periodic images. From a sufficiently large $L≳4\overset{{}^{\circ}}{A}$ distance, the H-atom in the supercell can be interpreted as a free non-interacting H-atom. By adding the atomic energy of the employed soft PAW potential for H ion, which causes an artificial constant shift in the absolute total energy.

The qualitative explanation behind this observation can be drawn from Figure 5: the majority of the 1*s* effective mass state are localized in the core region of the defect in which the localized orbitals are confined. As a consequence, the electron cloud of the localized orbitals will shield the effective attractive potential of the defect and repel the 1*s* effective mass state which finally shifts its energy level closer to the ionisation threshold energy. The quantative prediction of the 1*s* energy level calls for *ab initio* calculations. Unlike the case of the deep defects in Si with bound exciton excited states, the relatively short Bohr-diameter of the VBM hole 1*s* state makes it possible to embed the excited state wavefunction in a few-thousand atom supercell, viable at KS DFT level. From a remote distance, the SiV(−) + hole system looks completely neutral which is the case of a giant supercell by completely embeding the 1*s* wavefunction. In smaller sizes of supercells, the systems looks like a negatively charged defect as the hole wavefunction is completely delocalized within the applied supercells, and a total energy correction should be applied similar to the SiV(−) defect. However, the total energy correction for the SiV(−) + hole system should not be exactly the same with the SiV(−) defect as the size of the supercell is increased because the bound hole provides a screening towards the SiV(−) defect core. Thiering and Gali suggested the following formula [11],

(22) $$E_{n=1}^{\text{corr}}=\frac{A}{L}exp\left(-\frac{D}{L}\right)+\frac{B}{L^{3}}+C\text{,}$$

where *D* is the screening length which effectively screens the monopole charge induced by the defect, the *s*-type spherical potential. The quadropole term *B* may also incorporate the strain field effects too, and *C* is the value of the dilute limit. It is a critical issue how large could be the screening length. This can be illustrated by numerical modelling of a hydrogen atom in the simple cubic supercell with lattice constants (*L*) which can be calculated at Hartree–Fock level within VASP with using a soft PAW potential. The results are shown in Figure 7(d). One can clearly see that the Coulombic scaling (−1/*L*) deviates at sufficiently large supercells. At sufficiently large supercell size (*L* > 4 Å), the total energy of the system converges exponentially to a constant energy. The dilute limit (*C*) is found by

(23) $$E_{H}\left(L\right)=\frac{A}{L}\exp\left(-\frac{D_{H}}{L}\right)+C\text{,}$$

where the Coulomb interaction (*A*/*L*) is screened by $\exp\left(-\frac{D_{\text{H}}}{L}\right)$. We note that the repulsive 1/*L*^3^ term is missing because only a single proton appears in the system. Using this fitting procedure, $D_{\text{H}}=1.90\;\overset{{}^{\circ}}{A}=3.56\cdot a_{0}$ was found, where $a_{0}=0.53\overset{{}^{\circ}}{A}$ is the Bohr-radius of the isolated free hydrogen atom for *n* = 1 1*s* state.

One can conclude from the results of this simple model that the screening length is multiple times longer than the Bohr-radius of the effective mass state. This result could explain the need of simplified computational approaches on silicon defects described above because the Bohr-radius itself is already too long to be accommodated by 10,000 atom supercell, so the screening radius cannot be computed at *ab initio* level. In those cases, Eq. (21) was applied [20] which overcorrects because of the neglect of the screening effect. In diamond, the Bohr-radius of the effective mass hole states is short enough to observe the deviation from the formula in Eq. (21) due to the screening effect in few thousand atom supercell calculations. Thiering and Gali in Ref. [11] applied PBE functional to yield the 1*s* total energy by ΔSCF method including 8000-atom supercells, and the resulting screening length was fixed in the fit to HSE06 ΔSCF energies as a function of the supercell size where the maximum size was 1000-atom supercell (Figure 7(b)). The scalings of the charge transition level (Figure 7(a)) and the excitation energy of the 1*s* state show similarities in the range of small supercell size but a clear deviation can be observed for supercell size with >1000 atoms. That deviation is essential to obtain the accurate value, in good agreement with the experimental data. Certainly, the highly localized orbitals and the corresponding excitation energy converges much faster with the size of the supercell as demonstrated in (Figure 7(c)) which also holds for the crystal field splitting and spin–orbit coupling parameters too [11].

These recent findings cannot be found in the textbooks about semiconductor physics and can be considered as an extension of effective mass theory towards the excited states of deep defects.

The bound exciton states may be formed not just upon optical excitation but by capturing carriers by the deep defects. The capture rate can be, in particular, effective if the defect Coulombically attract the carriers. In the previous example, it can be imagined that the SiV(−) defect in its ground state captures a hole from VBM, which turns SiV(−) to SiV(0) plus a bound hole excited state, and that will decay either radiatively or non-radiatively to the ground state of SiV(0). This effect was first considered in the electroluminescence of single NV defect in diamond (see Supplemental Material in Ref. [99]). In this case, the negatively charged NV defect binds a hole, creating NV(−) plus bound hole from the VBM. That was calculated by HSE06 ΔSCF method without studying the convergence of the excitation energy [99]. In a later study [100], the interaction of two individual NV defects was investigated when the photo-ionisation of one NV leads to the emission of holes toward the neighbour NV(−) defect which can capture that hole. According to the interpretation of the measurements, a giant *σ*_*h*_ ≈ 3 × 10^−3^ μm^2^ hole capture rate was derived for NV(−). This was rationalised by involving the formation of a bound exciton state featuring an electron localized at the NV(−) plus a bound hole from VBM. Flick in Ref. [100] calculated the total energy of this bound exciton excited state by following the recipe in Ref. [11] with a little modification of Eq. (22) to apply the screening effect also on the quadrupole term. It was found that the binding energy of the exciton is about 40 meV for the 1*s* state [100]. That is definitely a stable state at room temperature.

### 3.4 Computation of zero-field splitting for high-spin defects and the *g*-tensor for defects with heavy ions

The computation of magneto-optic parameters of defect qubits such as zero-field splitting (ZFS) or *g*-tensor is of high importance not only because that they act as a fingerprint for identification of defect qubits with unknown microscopic structure but they can govern the type of interaction with external magnetic, electric, and strain fields as well as temperature. The importance of interaction between the hyperfine tensor with the strain field was already illustrated in Section 3.3.4. Here, we briefly list the advance in the calculation of ZFS and *g*-tensor for defect qubits.

The high-spin (*S* ≥ 1) defects with axial or lower symmetry may experience the electron spin-electron spin dipole-dipole interaction that may be expressed as

(24) $$\begin{array}{ll} H_{\mathrm{SS}} & =-\frac{\mu_{0}g_{e}^{2}\mu_{B}^{2}}{4\pi }\sum_{i\;>j}\frac{3\left({\hat{S}}_{i}r_{ij}\right)\left(r_{ij}{\hat{S}}_{j}\right)-\left({\hat{S}}_{i}{\hat{S}}_{j}\right)r_{ij}^{2}}{{\left|r_{ij}\right|}^{5}}\\ & \equiv \sum_{i\;>j}{\hat{S}}_{i}D_{ij}{\hat{S}}_{j}\text{,} \end{array}$$

where **r**_*ij*_ = **r**_*i*_ − **r**_*j*_. The 3 × 3 *D*-tensor can be diagonalized to find the spectrum and spin eigenstates. The *D* tensor is associated with the two-particle spin density matrix, *n*_2_(*r*_1_, *r*_2_), which can be approximated by using the Slater-determinant of the KS wave functions *ϕ* of the considered system, so that $n_{2}\left(r_{1},r_{2}\right)\approx {\left|\Phi_{ij}\left(r_{1},r_{2}\right)\right|}^{2}$, where $\Phi_{ij}\left(r_{1},r_{2}\right)=\frac{1}{\sqrt{2}}\left(\phi_{i}\left(r_{1}\right)\phi_{j}\left(r_{2}\right)-\phi_{j}\left(r_{1}\right)\phi_{i}\left(r_{2}\right)\right)$ and then

(25) $$\begin{array}{ll} D_{ab} & =\frac{1}{2}\frac{\mu_{0}}{4\pi }\frac{g_{e}^{2}\mu_{B}^{2}}{S\left(2S-1\right)}\overset{\text{occupied}}{\underset{i\;>j}{\sum }}\chi_{ij}\\ & \;\times \int {\left|\Phi_{ij}\left(r_{1},r_{2}\right)\right|}^{2}\frac{r^{2}\delta_{ab}-3r_{a}r_{b}}{r^{5}}d^{3}r_{1}d^{3}r_{2}\text{,} \end{array}$$

where $r_{a,b}={\left(r_{1}-r_{2}\right)}_{a,b}$ and *χ*_*ij*_ is either 1 or −1 for KS *i*, *j* states of the same or different spin channels, respectively. Note that in DFT the spin-polarised KS states are not spin restricted. Consequently, not only the unpaired KS states but also the rest of the occupied states can contribute to the spin density and the ZFS [131]. However, there is no guarantee in spin-polarised KS DFT methods that the final solution will be the eigenstate of the spin operator, and the discrepancy in it is called spin contamination. In practice, spin contamination may be small with using (semi)local DFT functionals but could be significant with hybrid DFT functionals. This may result in a significant error in the approximation of the two-particle spin density matrix. Biktagirov and co-workers suggested a workaround for this problem [132] which is illustrated for the *S* = 1 case. The idea is that if there is a spin contamination in the electronic structure then the *m*_*s*_ = 0 spin configuration with spin-polarised DFT should produce non-zero contribution to the *D*-tensor, called ${\bar{D}}_{m_{s}=S-1}$. The bare *D*-tensor with *m*_*s*_ = *S* spin-polarised DFT results in $D_{m_{s}=S}$. Finally, the corrected *D*-tensor, $\tilde{D}$, is

(26) $$\tilde{D}=\frac{S}{2}\left(D-\bar{D}\right)\text{.}$$

Each of these *m*_*s*_ = *S* − 1 configurations can be obtained by changing the occupation of one of the half-filled KS orbitals from spin up to spin down and subsequently performing the self-consistent field calculation. It was found that for divacancy defects in 4H SiC that the calculated *D*-constants are at around 1.6 GHz but $\tilde{D}=1.3$ GHz are obtained after correction, close to the experimental data (see Ref. [132] and references therein).

The ZFS may have other contribution for *S* ≥ 1 systems than electron spin-electron spin dipole-dipole (*D*_SS_) interaction as given in Eq. (25). As an example, we mention here the neutral nickel-vacancy (NiV) defect in diamond which has the same structure as SiV(0) defect discussed above in this review paper. NiV(0) has also ^3^*A*_2*g*_ ground state similar to that of SiV(0) with six carbon dangling bonds which constitute of the ground state electron wavefuntion. The calculated *D*_SS_ = 967 MHz = 0.004 meV which is typical for the third neighbour distance of dangling bonds in diamond (see Table 2 below). However, this value is very far from the observed *D* = 170 GHz = 0.703 meV (see Ref. [133] and references therein). As ^3^*A*_2*g*_ is an orbital singlet, thus first-order spin–orbit interaction does not enter here. However, second-order spin–orbit interaction between the ground state triplet and excited state singlet states may play a role which can be selective towards the *m*_*s*_ = 0 state of the triplet, and it results in an effective energy shift of the *m*_*s*_ = 0 level and opening the gap between the *m*_*s*_ = 0 and *m*_*s*_ = ±1 levels. To illustrate this using the first-order perturbation theory, we consider the interaction of the ^3^*A*_2*g*_ and ^1^*A*_1*g*_ state that are linked by the parallel component of the spin–orbit operator $\left({\hat{H}}_{\text{SO}}\right)$, *λ*_*z*_, where

(27) $${\hat{H}}_{\text{SO}}=\underset{i}{\sum }\lambda_{⊥}\left({\hat{L}}_{i,x}{\hat{S}}_{i,x}+{\hat{L}}_{i,y}{\hat{S}}_{i,y}\right)+\lambda_{z}{\hat{L}}_{i,z}{\hat{S}}_{i,z}\text{.}$$

**Table 2:** Spin–orbit and spin-spin contributions to the ZFS (in MHz) of the NV centre and a set of group-IV–vacancy defects in diamond (compared to available experimental data). All the defects have *S* = 1 ground state with orbital singlet *A*_2_-type many-body wavefunction. The geometry and electronic structure of group-IV–vacancy defects are akin to those of SiV(0). See Ref. [132] and references therein.

Defect	*D*_SO_	*D*_SS_	*D*_SO+SS_	Experiment
NV(−)	6	2722	2728	2878
SiV(0)	480	570	1050	929
GeV(0)	1469	630	2099	2248
SnV(0)	10,763	630	11,393	
PbV(0)	144,860	660	145,520	

The energy gap between ^3^*A*_2*g*_ and ^1^*A*_1*g*_ is Θ before applying ${\hat{H}}_{\text{SO}}$ and the spin–orbit coupling between |^3^*A*_2*g*_, *m*_*s*_ = 0⟩ and ^1^*A*_1*g*_ through *λ*_*z*_ is *λ*_0_. In that case, the first-order perturbation theory yields that |^3^*A*_2*g*_, *m*_*s*_ = 0⟩ level shifts downwards by $\lambda_{0}^{2}/\Theta$. This means that

(28) $$D_{\text{SO}}=\frac{\lambda_{0}^{2}}{\Theta }$$

and it will be dominant over *D*_SS_. According to HSE06 calculations (see details in Ref. [133]), Θ ≈ 0.68 eV and *λ*_0_ = 23.2 meV which results in *D*_SO_ = 0.79 meV. This is much closer to the experimental data at 0.703 meV.

One can go beyond the first-order perturbation theory and consider the change in the wavefunction due to spin–orbit interaction (second-order perturbation),

(29) $${\left|\Psi \right\rangle }^{\left(1\right)}={|}^{3}A_{2g},m_{s}=0)〉+\frac{\lambda_{0}}{\Theta }{|}^{1}A_{1g}〉\text{,}$$

which may result in a more accurate result than that by first-order perturbation theory.

In general, the problem can be rephrased by considering the total energy of the system as a function of the spin quantisation direction, $E_{\text{tot}}\left({\vec{n}}_{\sigma }\right)$. In a uniaxialrt: case, the magnetic anisotropy energy is then defined as difference between $E_{\text{tot}}\left({\vec{n}}_{\sigma }\right)$ obtained with ${\vec{n}}_{\sigma }$) parallel (*z*) and perpendicular (⊥) to the anisotropy direction, *E*_SO_ = *E*_tot_(*z*) − *E*_tot_(⊥), and then the corresponding *D*-constant can be evaluated as *D*_SO_ = *E*_SO_/*S*^2^ for integer *S* and *D*_SO_ = *E*_SO_/(*S*^2^ − 1/4) for half-integer *S*. For the case of NiV(0) with *S* = 1, *E*_SO_ should be calculated self-consistently. *E*_tot_(*z*) = *E*_tot_(↑↑) calculation can be carried out with the usual *m*_*s*_ = +1 setting for the KS orbitals together with the scalar-relativistic spin–orbit interaction. *E*_tot_(⊥) calculation is a bit tricky. The two spins should be individually rotated by 90° about the *x*-axis. Then the total energy of the system should be calculated by scalar-relativistic spin–orbit interaction. This total energy is not identical to *E*_tot_(⊥) because the rotated-spins system is not the exact *m*_*s*_ = 0 eigenstate but a mixture of *m*_*s*_ = ±1 and *m*_*s*_ = 0, so we label it by *E*_tot_(→ →). The final expression [133] is

(30) $$D_{\text{SO}}=3\left(E_{\text{tot}}\left(↑↑\right)-E_{\text{tot}}\left(\to \to \right)\right)\text{.}$$

The self-consistent HSE06 *D*_SO_ = 0.73 meV which is 0.06 meV deeper than the first-order perturbation theory value at 0.79 meV, and it brings the result closer to the experimental data at 0.703 meV. This shows that self-consistent spin–orbit calculation needed for obtaining accurate ZFS for defects consisting of heavy ions. It is interesting to note that self-consistent spin–orbit PBE calculations results in *D*_SO_ = 1.35 meV which is significantly larger than the HSE06 and experimental values. We note that Θ ≈ 0.25 eV with PBE which explains the too large *D*^SO^ with PBE as first-order perturbation theory showed that *D*^SO^ scales inversely between the gap of the triplet and singlet levels [133].

These results clearly demonstrate [133] that the energy gap between triplet (high-spin) and singlet (low-spin) levels are highly critical in obtaining an accurate ZFS for defects which consist of heavy ions.

The disasvantage of the self-consistent spin–orbit calculations is that it can principally work for sufficiently large spin–orbit energies, usually created by heavy atoms, so that it does not fall below the numerical noise. For defects with light atoms, one has to rely on the first-order perturbation theory which was previously sketched for a special case (diamond NiV defect) as an introduction to the problem.

Biktagirov and co-workers [134] implemented the perturbation theory based method to the GIPAW-tree of the Quantum Espresso package. They apply collinear spin polarisation approximation with direction *a* = *x*, *y*, *z*. Then the SO coupling in direction *a* $\left({\hat{H}}_{a}^{\text{SO}}\right)$ and *b* $\left({\hat{H}}_{b}^{\text{SO}}\right)$ contributes to the total energy of the system in second-order perturbation theory as

(31) $$E_{ab}^{\text{SO}}=\underset{o,s,s^{\prime }}{\sum }\text{Re}\left\langle {\bar{\Psi }}_{o}^{s}\left|{\hat{H}}_{a}^{SO}G^{s^{\prime }}\left(\epsilon_{e}\right){\hat{H}}_{b}^{SO}\right|{\bar{\Psi }}_{o}^{s}\right\rangle \text{.}$$

Here the sum runs over the spin channels *s* and *s*′ and the occupied states *o* ∈ *s*. Thereby, $|{\bar{\Psi }}_{o}^{s}〉$ are the corresponding unperturbed KS wave functions (obtained without SO coupling), and *G*^*s*^^′^(*ϵ*_*e*_) is the Green’s function of the empty states *e* ∈ *s*′. In their implementation, explicit summation over empty states is avoided by calculating *G*^*s*′^(*ϵ*_*e*_) by projecting the empty states onto the valence bands. By this, the approach becomes faster, numerically more stable, and almost unaffected by the gap issues quoted above for the (semi)local DFT functionals. On the other hand, the method is not so intuitive as the majority of the interaction comes from the closest high-spin low-spin energy states which cannot be directly analysed by this method.

Biktagirov and co-workers applied their method to diamond NV centre and group-IV–vacancy defects [134]. The results are listed in Table 2. As can be seen the defect with the lightest atom exhibits the smallest *D*_SO_ whereas it increases orders of magnitude with going to heavier atoms. In the group-IV–vacancy defects *D*_SS_ increases slightly as the heavier atoms push the neighbour carbon atoms farther from each other but the vast contribution comes from *D*_SO_ except for SiV(0) and partially for GV(0) where the two contributions are similar. We note that PbV(0) shows about *D*_SO_ = 145 GHz for which self-consistent *D*_SO_ calculation would result in a lower value.

Although, the calculated *D*_SO_ is only 6 MHz for diamond NV centre but it couples directly to the electric field unlike *D*_SS_ which couples to the electric field only indirectly via changing the electron cloud so the spin density (e.g., Ref. [135]). As a consequence, the field applied along the defect’s symmetry axis, the *D*_SO_ part dominantly drives the predicted Stark coefficient, 0.034 GHz Å/V, into the experimentally observed confidence interval of 0.035 ± 0.002 GHz Å/V (see Ref. [134]). The simulation was carried in a (111) diamond slab where the electric field was switched on during the calculations of *D*_SS_ and *D*_SO_.

Previously, we discussed the spin level structure in the absense of external magnetic field. Nevertheless, it is highly important to understand the coupling of defect spins to external magnetic fields. The external magnetic fields could be intentionally turned on for manipulation of the qubits on one hand, and on the other hand, randomly distributed electron or nuclear spins proximate to the defect qubits could influence their longitudonal relaxation and coherence times. Here, we discuss the issue of a constant macroscopic external magnetic field interacting with the defect’s electron spin which can be generally described as

(32) $$\hat{H}=-B\hat{\mu }=B\mu_{\text{B}}g\tilde{S}\text{,}$$

where $\hat{\mu }$ is the magnetic dipole momentum operator, *μ*_B_ is the Bohr-magneton of the electron, **g** is the *g*-factor, and $\tilde{S}$ is a phenomenological pseudo-spin, which is set to the net electron spin of the system, i.e., $\tilde{S}=1/2$ for the Kramers-doublet defect’s electron spin. Eq. (32) has the form of the Zeeman formula for the free electron but *g*_*e*_ = 2.0023 free electron scalar value is substituted by **g**. Unlike the free electron case, the defect’s electron spin feels the potential of ions which is less symmetric than spherical, the defect’s electron may have an effective angular momentum with this condition, e.g., localized on the *d*-orbital, which can be also influenced by the electron-phonon coupling. All of these effects are packed into a single tensor **g**.

This issue is illustrated on the neutral vanadium defect substituting the Si-site in 4H SiC which has become a very promising spin-to-photon interface with a quantum memory and optical emission at the telecom wavelength (see Refs. [136, 137] and references therein). The *d*-orbital of the vanadium ion splits due to the C_3*v*_ symmetric crystal field of 4H SiC and then a double degenerate *e*-orbital occurs in the gap localized on the *d*-orbital of vanadium, occupied by a single electron. Because of the double degenerate *d*-orbital, one can expect an effective spin–orbit coupling between the orbital and the electron spin, where the low symmetry crystal field will reduce the effective angular momentum of the orbital called Stevens reduction factor (*r*) (e.g., see the origin of this effect in more detail for group-IV–vacancy defects in Ref. [138]). However, it is also known that this is an *E* ⊗ *e* Jahn–Teller system [139, 140] which can also effectively reduce the angular momentum of the electron orbit so the effective spin–orbit splitting known as Ham reduction factor (*p*). As a consequence, $\hat{\mu }$ in Eq. (32) can be written as

(33) $$\hat{\mu }=-\left(\mu_{\text{B}}pr\hat{L}+\mu_{\text{B}}g_{e}\hat{S}\right)=-\mu_{\text{B}}g\tilde{S}\text{,}$$

where the contributions of $\hat{L}$ and $\hat{S}$ are separated, so it gives an opportunity to unravel the microscopic origin of **g**. Csóré and Gali carried out *ab initio* calculations [141] to determine the *r* and *p* factors, so then **g** can be obtained as

(34) $$g_{\|}=2\left(g_{e}S_{z}+L_{z}^{\text{eff}}\right)=\frac{2\mu_{z}}{\mu_{B}}\text{,}$$

(35) $$g_{⊥}=\frac{\mu_{+}+\mu_{-}+i\left(\mu_{-}-\mu_{+}\right)}{\mu_{B}}\text{,}$$

where expectation values of the ladder magnetic dipole moment operators are used (*μ*_±_) to express *g*_⊥_. In Eq. (34) *S*_*z*_ and $L_{z}^{\text{eff}}$ are expectation values of ${\hat{S}}_{z}$ and the effective angular momentum operator, ${\hat{L}}_{z}^{\text{eff}}=pr{\hat{L}}_{z}$, respectively.

We note that the *d*-orbitals may require a special attention for accurate calculation, and indeed, HSE06 DFT functional overlocalises the *d* state that should be corrected [142]. For the heavy-atom defects one may assume that the vast majority of the spin–orbit coupling, so the effective angular momentum, comes from the single heavy-atom. In this case, the analysis of the *d*-orbitals and the actual DFT wavefunctions can reveal the deviation of the *d*-orbitals from the spherical symmetry (see Table 3), so the *r* can be computed. As an example, Ψ_1_ and Ψ_4_ as well as Ψ_2_ and Ψ_3_ can be coupled in Eq. (34) where the corresponding wavefunction coefficients can be extracted for the given spin–orbit state in the KS DFT calculation. It was found that the vanadium at one site of 4H SiC feels isotropic environment with small effective angular momentum which is an order of magnitude larger in a truly axial-like environment at the other site of 4H SiC. After solving the Jahn–Teller Hamiltonian (see Refs. [30, 141]), the typical Ham reduction factor is at about 0.6 which is significant, so the electron-phonon coupling effectively reduce the interaction between the defect’s pseudo-spin and the external magnetic field parallel to the symmetry axis of the defect. The final typical values of *g*_‖_ are around 1.9.

**Table 3:** Kramers doublets formed by *d*-orbitals and the corresponding single and double group irreducible representations under C_3v_ symmetry. We give widespread notations for double group irreducible representations (irreps) and also the corresponding *m*_*j*_ = *m*_*l*_ + *m*_*s*_ values.

Labels	Orbitals	Irreps.		*m*_*j*_
		Single	Double	
Ψ_1_	$|d_{+2},+\frac{1}{2}〉;|d_{-2},-\frac{1}{2}〉$	^2^ *E*	$E_{\frac{1}{2}}$ (Γ_4_)	$\pm \frac{5}{2}$
Ψ_2_	$|d_{+2},-\frac{1}{2}〉;|d_{-2},+\frac{1}{2}〉$	^2^ *E*	$E_{\frac{3}{2}}$ (Γ_5,6_)	$\pm \frac{3}{2}$
Ψ_3_	$|d_{+1},+\frac{1}{2}〉;|d_{-1},-\frac{1}{2}〉$	^2^ *E*	$E_{\frac{3}{2}}$ (Γ_5,6_)	$\pm \frac{3}{2}$
Ψ_4_	$|d_{+1},-\frac{1}{2}〉;|d_{-1},+\frac{1}{2}〉$	^2^ *E*	$E_{\frac{1}{2}}$ (Γ_4_)	$\pm \frac{1}{2}$
Ψ_5_	$|d_{0},+\frac{1}{2}〉;|d_{0},-\frac{1}{2}〉$	^2^ *A*_1_	$E_{\frac{1}{2}}$ (Γ_4_)	$\pm \frac{1}{2}$

For the calculation of *g*_⊥_ (Eq. (35)), the ladder magnetic dipole operator was considered, ${\hat{\mu }}_{\pm }$ that can couple state |*m*_*j*_⟩ to state |*m*_*j*_ ± 1⟩, where *m*_*j*_ = *m*_*l*_ + *m*_*s*_. However, the in-gap defect states transform as either *E*_1/2_ (linear combination of Ψ_1_ and Ψ_4_) or *E*_3/2_ (linear combination of Ψ_2_ and Ψ_3_) with the *m*_*j*_ values given in Table 3. Consequently, ${\hat{\mu }}_{\pm }$ cannot couple neither Ψ_1_ and Ψ_4_, nor Ψ_2_ and Ψ_3_ therefore *g*_⊥_ = 0 in each case. Deviation from *g*_⊥_ = 0 may occur due to secondary effects.

The same method was applied to the nickel defects in diamond where the NiV(−) was identified by first principles calculations as an excellent qubit candidate analogous to the group-IV-vacancy qubits in diamond [133] which has an optical emission at about 1.4 eV and a highly anisotropic *g*-tensor. In the literature, the NE4/AB1 EPR centre with *S* = 1/2 spin and relatively isotropic *g*-tensor with *g*_‖_ = 2.0027(2) and *g*_⊥_ = 2.0923(2) was previously associated with NiV(−) which is linked to the 1.72-eV optical centre (see Ref. [143] and references therein). Clearly, the NE4/AB1 centre should be associated with another nickel-related defect in diamond. Thiering and Gali tentatively assigned Ni_s_(N_s_)_3_(0) defect to this centre which has an unpaired electron on the *a*_1_ orbital strongly localized on Ni 3*d* orbitals. In this case, the *g*-tensor is modified from the free electron value because of the orbital moment of the Ni 3*d* states as explained above for vanadium defects in 4H SiC. This justifies to calculate the total orbital moment $\left(\left\langle {\hat{L}}_{x,y,z}\right\rangle \right)$ of the defect within the PAW sphere of the ions where the largest contribution comes from the Ni ion. Because of C_3*v*_ symmetry $\left\langle {\hat{L}}_{x}\right\rangle =\left\langle {\hat{L}}_{y}\right\rangle =\left\langle {\hat{L}}_{⊥}\right\rangle$. The main components of the *g*-tensor can be given as $g_{\|}=g_{e}+2\left\langle {\hat{L}}_{z}\right\rangle$ and $g_{⊥}=g_{e}+2\left\langle {\hat{L}}_{⊥}\right\rangle$. Finally, *g*_‖_ = 2.0058 and *g*_⊥_ = 2.0942 are obatined, in good agreement with the experimental data.

### 3.5 Spin-phonon coupling: temperature-dependent longitudonal spin relaxation time and magneto-optical parameters

#### 3.5.1 Longitudonal spin relaxation time

A key parameter of qubits is the longitudonal spin relaxation time which is the characteristic time of flipping the spin, and it is labeled by *T*_1_ in the literature. This sets the absolute limit of the spin coherence time, i.e., the characteristic quantum information processing operation time of the qubit. It is of high importance to understand the underlying microscopic processes. In nuclear spin physics, the origin of spin flipping was identified as the interaction between phonons and the spin which is manifested as a highly temperature-dependent phenomenon; therefore, it is also often called spin-lattice relaxation time. As *T*_1_ often exponentially decay with elevating the temperature it is imperative to characterise *T*_1_ as a function of temperature, in order to explore the applicability of qubits as sensor probes in biology which requires ambient conditions. Our review paper only focusses to the recent advances on defect qubits, in particular, on *S* ≥ 1 defect qubits.

Spin flipping processes require such interaction Hamilton operator which contains spin shift operators. It can be easily recognized that the spin-spin dipole-dipole interaction in Eq. (24) contains single and double spin shift operators, e.g., ${\hat{S}}_{x}{\hat{S}}_{z}$ and ${\hat{S}}_{x}{\hat{S}}_{x}$, respectively. Therefore, if the defect qubit’s spin interact with other spin species then it causes a spin flip of the defect qubit. The strength of dipole-dipole interaction goes with inverse cube of the distance between the spins. This interaction is weakly dependent from temperature and it highlights that the longitudonal spin relaxation time is not necessarily a spin-phonon interaction. In practice, this type of *T*_1_ process becomes only important at elevated temperatures for diamond NV centre if the concentration of defect spins is relatively high, e.g., 4–8 particle per million (ppm) in diamond [62].

Ivády applied the cluster-correlation expansion (CCE) [144, 145] to model the interaction of the central diamond NV centre’s electron spin with other electron spins such as the environmental NV centres, nitrogen donor spins (labelled as P1 EPR centre), and the ^13^C nuclear spins also as a function of the external magnetic field and strain [62]. The CCE approach will be shortly discussed in the next chapter.

We note that another study only restricted this investigation to the bath of ^13^C nuclear spins but taking only the dipolar interactions into account with far ^13^C nuclear sites [146]. However, the bath of ^13^C with Fermi-contact hyperfine terms cannot be ignored for accurate simulations which calls for *ab initio* simulations.

Ivády calculated the hyperfine tensors for ^13^C isotopes by HSE06 DFT method in a 1728-atom simple cubic supercell [62]. Since the hyperfine tensors should be determined at large distances from the defect site this required a special approach in order to avoid finite size effect problems. Ivády utilised a real space grid combined with the PAW method to calculate hyperfine tensors. The Fermi contact term, dipole-dipole interaction within the PAW sphere, and core polarisation corrections are calculated within the PAW formalism from the convergent spin density. The dipolar hyperfine contribution from spin density localized outside the PAW sphere is calculated by using a uniform real space grid. This procedure enabled to obtain hyperfine coupling tensors excluding effects from periodic replicas of the spin density due to the periodic boundary condition. Additionally, hyperfine tensors for atomic sites outside the boundaries of the supercell were calculated by neglecting Fermi contact interactions with achieving a smooth transition in the hyperfine constants at the boundary of the two approaches.

Ivády used the extended Lindbladian equation in order to simulate the spin dynamics of the central spin and its relaxation rate 1/*T*_1_ in materials where the electron spin density cannot be ignored beside the nuclear spin bath [62]. In this model the total spin relaxation rate (1/$T_{1}^{\text{tot}}$) is

(36) $$\frac{1}{T_{1}^{\text{tot}}}=\frac{1}{T_{1}^{\text{P}1}}+\frac{1}{T_{1}^{\text{NV}-\text{basal}}}+\frac{1}{T_{1}^{\text{NV}-\text{par}}}+\frac{1}{T_{1}^{13\text{C}}}\text{,}$$

where “NV-basal” and “NV-par” label such NV centres in the environment which have other and parallel symmetry axis with that of the central NV centre. Finally, it was found that the environmental NV centres have a dominant effect on the spin relaxation rate [62]. At special setting of the magnetic fields, either ground state level anticrossing (GSLAC) or excited state level anticrossing (ESLAC), the relaxation rate is accelerated because the P1 centres and the nuclear spins can easily induce spin flip-flop processes that were otherwise protected by the energy gap between the electron spin levels of the NV centre. At GSLAC, the spin-polarisation of the electron spin and the coupled nuclear spin also changes that can be observed by the change of the PL intensity as the external magnetic field is swept around the GSLAC region [147]. This modelling also rationalised the photo-electric read-out process of the single ^14^N nuclear spin of the NV centre at ESLAC condition [148].

$T_{1}^{\text{P}1}$ time was further investigated in detail [149] in which they considered the microscopic structure of the P1 centres in diamond, namely, the strong Jahn-Teller distortion will generate four different symmetry ⟨111⟩ axes in diamond, and the ^14^N nitrogen hyperfine tensor to the P1 centre’s electron spin adapts to these orientations. As a consequence, the spin flip-flop processes between the P1 pairs are reduced with respect to the case of unrealistic aligned P1 centres [149]. With taking the microscopic structure of the P1 centres into account, the calculated spin relaxation times of the diamond NV centre exhibits a clear linear dependence on P1 concentrations on a log scale with a slope of −1.06, in excellent agreement with some experimental data (see Ref. [149] and references therein).

This theory was also applied to the divacancy qubit in 4H SiC by considering other divacancy spins (*S* = 1), negatively charged Si-vacancy spins (*S* = 3/2), nitrogen donor spins (*S* = 1/2), as well as ^13^*C* and ^29^Si *I* = 1/2 nuclear spins in the environment, also as a function of the external magnetic field [150]. It was found that the cross-relaxation accelerate spin flip-flop rates again in the region of GLSAC and ESLAC magnetic fields for each considered environmental spins. At zero magnetic field a simple relation was found for the interaction between N-donor and the central divacancy spin,

(37) $$\frac{1}{T_{1}}=\beta C^{2}\text{,}$$

where *β* = 1.6 × 10^−35^ Hz/cm^−6^ and *C* is the concentration of the N-donor. It is noted that nitrogen implanted samples the distribution of nitrogen donor is not homogeneous, and then the “concentration” should be considered near the target divacancy spin which was created as a result of the implantation [150]. The theory was also employed to the Si-vacancy *S* = 3/2 qubit in 4H SiC [151]. In this case, Bulancea-Lindvall and co-workers considered the interaction the Si-vacancy qubit spin with *S* = 1/2 defects, e.g., N-donors. Si-vacancy in 4H SiC has minor ZFS, thus at a given external magnetic field with similar Zeeman shifts the two spin systems can be effectively coupled by dipole-dipole interaction unlike the case of divacancy with *S* = 1 spin and high ZFS (≈1.3 GHz). On the other hand, ^29^Si nuclear spins at natural abundance (4.5%) exhibit a considerable hyperfine splitting when they interact with the Si-vacancy qubit spin which will supress the cross-relaxation between the Si-vacancy qubit spin and the *S* = 1/2 spins in the environment. By isotope purification (reducing the concentration of ^29^Si isotopes in 4H SiC), the cross-relaxation so the spin flip-flop process accelerates and the spin relaxation time of the Si-vacancy qubit significantly reduces when the concentration of *S* = 1/2 spins are high ($\approx 10^{15}-10^{18}$ cm^−3^). This surprising result was unraveled by these simulations with using *ab initio* hyperfine tensors in the parametrisation of the interaction Hamiltonian.

High quality materials with single defect spins and low concentration of nuclear spins do not experience spin flip-flop events with electron spins in the environment, and the flip-flop processes caused by nuclear spins are only observable at special conditions (e.g., external magnetic field is set to GSLAC condition). In this case, the spin-phonon coupling is responsible for *T*_1_ and it becomes strongly temperature dependent. In the case of molecules, vibrations are indeed responsible behind the spin flipping process. In a recent review paper [152], the *ab initio* theory and its application to molecules have been presented in detail. The formulas and basic equations apply to defect qubits too which are not reiterated here in detail.

Regarding the temperature dependence of *T*_1_ = 1/Γ of defect qubits, the most studied one is the diamond NV centre [31, 153], [154], [155], and the first *ab initio* results have been reported for this defect qubit because the theories could be well tested on the accurately recorded experimental data. In recent studies [31, 156], the *m*_*s*_ = ±1 levels of ^3^*A*_2_ ground state was split by a small external magnetic field aligned parallel to the symmetry axis of the defect (≈145 MHz), and they could observe both single-flip rates (|0⟩ ↔ |±1⟩, labelled by Ω) and double-flip rates (|+1⟩ ↔ |−1⟩, labelled by *γ*) and found that *γ* > Ω at any observed temperature (*T* > 125 K) and *γ* ≈ 2 Ω at room temperature. In the temperature region of 125 K and 400 K, the rates increase from ≈1 Hz to ≈200 Hz. At this temperature region only phenomenological theory was considered using an empirical model in which the high-temperature behavior is characterized by a term that scales with temperature as *T*^5^ [153, 154], which may arise due to Raman scattering of low-energy acoustic phonons which are weakly coupled to the spin via first-order interactions. However, insights from *ab initio* simulations should verify this model including the magnitude of the double-flip rates.

The spin relaxation rate may be expressed as

(38) $$\Gamma =\Gamma_{0}+\Gamma_{1}^{\left(1\right)}\left(T\right)+\Gamma_{1}^{\left(2\right)}\left(T\right)+\Gamma_{2}^{\left(1\right)}\left(T\right)+\ldots \text{,}$$

where the superscript refers to the order of the spin-phonon interaction (i.e., terms with superscript 1 or 2 are linear or quadratic in the atom displacements respectively) and the subscript refers to the order in perturbation theory. Γ_0_ is a sample-dependent constant term arising from spin-spin interactions that was discussed above. $\Gamma_{1}^{\left(1\right)}$ describes the absorption or emission of a single resonant phonon by the spin. Because the ZFS energy of the NV ground-state triplet is small in comparison to typical phonon energies in diamond, this process is only relevant at subkelvin temperatures [155]. $\Gamma_{2}^{\left(1\right)}$ corresponds to the Raman-scattering of low-energy acoustic phonons via first-order interactions. However, we will present below [156] unlike for other spin systems (e.g., several coordination compounds as recently shown in Ref. [157]), first-order spin-phonon interactions provide only negligible contributions to Raman scattering for the NV centre in diamond. The major effect comes from $\Gamma_{1}^{\left(2\right)}\left(T\right)$ so the quadratic displacements of ions.

In order to calculate $\Gamma_{1}^{\left(2\right)}\left(T\right)$, the spin-phonon matrix elements should be obtained by exploiting the dependence of the *D*-tensor on the normal coordinates (*Q*) as

(39) $$\begin{array}{ll} \overset{⃡}{D}\left(R\right) & =\overset{⃡}{D}\left(R=0\right)+{\left.\underset{i}{\sum }\frac{\partial \overset{⃡}{D}}{\partial Q_{i}}\right|}_{R=0}Q_{i}\\ & {\left.\;+\frac{1}{2}\underset{ij}{\sum }\frac{\partial \overset{⃡}{D}}{\partial Q_{i}\partial Q_{j}}\right|}_{R=0}Q_{i}Q_{j}\text{,} \end{array}$$

where the coefficients in Eq. (39) were extracted from VASP PBE calculations as implemented by Thiering and Gali [156]. In order to evaluate the second-order derivatives, only the diagonal terms were considered which satisfy *i* = *j* and distort the *C*_3*v*_ symmetric atomic positions by all degenerate *e*_*x*_, *e*_*y*_ phonon modes of the supercell by $\sqrt{{\left(\Delta R\right)}^{2}}=0.1\;\text{Å}\sqrt{\text{a.m.u.}}$. The second-order spin-flipping matrix elements $V_{+0}^{ll}$ and $V_{+-}^{ll}$ then determine the *D*-tensor according to the symmetry-adapted expression in which only the quadratic terms are expresse here as

(40) $$\begin{array}{ll} & \left(\begin{array}{ccc} -\frac{1}{3}\\ & -\frac{1}{3}\\ & & +\frac{2}{3} \end{array}\right)\underset{i}{\sum }3V_{00}^{ii}R_{i}^{2}+\underset{l}{\sum }V_{+-}^{ll}\\ & \;\times \left[\left(\begin{array}{cc} 1\\ & -1 \end{array}\right)\left(X_{l}^{2}-Y_{l}^{2}\right)+\left(\begin{array}{cc} & 1\\ 1 \end{array}\right)2X_{l}Y_{l}\right]\\ & \;+\underset{l}{\sum }\sqrt{2}V_{+0}^{ll}\left[\left(\begin{array}{ccc} & & 1\\ 1 \end{array}\right)\left(X_{l}^{2}-Y_{l}^{2}\right)\right.\\ & \;\left.+\left(\begin{array}{ccc} & & 1\\ & 1 \end{array}\right)2X_{l}Y_{l}\right] \end{array}$$

where *R*_*i*_, *X*_*l*_ and *Y*_*l*_ are the dimensionless coordinates (*not normal coordinates*) for the phonon modes at energy *ℏω*_*i*_ or *ℏω*_*l*_. We note that while the index *l* only covers the *e* modes once, the index *i* covers all *a*_1_, *a*_2_, *e*_*x*_, *e*_*y*_ modes and thus runs over the *e* modes twice. Eq. (40) can be employed to transform it into the spin-phonon interaction $\hat{V}$. By expicitly writing only the quadratic terms, it reads as

(41) $$\begin{array}{ll} \hat{V} & =\overset{⃖}{S}\;\overset{⃡}{D}\;\vec{S}\\ & =D\left({\hat{S}}_{z}^{2}-\frac{1}{3}S\left(S+1\right)\right)\\ & \;+\underset{i}{\sum }3V_{00}^{ii}\left({\hat{S}}_{z}^{2}-\frac{1}{3}\hat{S}\left(\hat{S}+1\right)\right){\hat{R}}_{i}^{2}\\ & \;+\underset{l}{\sum }V_{+-}^{ll}\left[\left({\hat{S}}_{x}^{2}-{\hat{S}}_{y}^{2}\right)\left({\hat{X}}_{l}^{2}-{\hat{Y}}_{l}^{2}\right)\right.\\ & \;\left.+\left({\hat{S}}_{x}{\hat{S}}_{y}+{\hat{S}}_{y}{\hat{S}}_{x}\right)\left({\hat{X}}_{l}{\hat{Y}}_{l}+{\hat{Y}}_{l}{\hat{X}}_{l}\right)\right]\\ & \;+\underset{l}{\sum }\sqrt{2}V_{+0}^{ll}\left[\left({\hat{S}}_{x}{\hat{S}}_{z}+{\hat{S}}_{z}{\hat{S}}_{x}\right)\left({\hat{X}}_{l}^{2}-{\hat{Y}}_{l}^{2}\right)\right.\\ & \;\left.+\left({\hat{S}}_{y}{\hat{S}}_{z}+{\hat{S}}_{z}{\hat{S}}_{y}\right)\left({\hat{X}}_{l}{\hat{Y}}_{l}+{\hat{Y}}_{l}{\hat{X}}_{l}\right)\right]\text{,} \end{array}$$

where $\overset{⃖}{S}={\left(\vec{S}\right)}^{†}=\left(\begin{array}{ccc} {\hat{S}}_{x} & {\hat{S}}_{y} & {\hat{S}}_{z} \end{array}\right)$. The dimensionless coordinates are expanded in terms of the phonon creation and annihilation operators: ${\hat{R}}_{i}=\left(b_{i}^{†}+b_{i}\right)/\sqrt{2}$ and ${\left\{\hat{X},\hat{Y}\right\}}_{l}=\left(b_{\left\{X,Y\right\}l}^{†}+b_{\left\{X,Y\right\}l}\right)/\sqrt{2}$.

As a next step for defining the equations for the rates, one can apply RPA for these processes so it is assumed that the consequtively absorbed phonons are not coherent. Furthermore, one can further simplify the equations by considering the fact that the ZFS energy of the diamond NV centre is much smaller than the typical phonon energies coupled to the spin. The key matrix elements are the first-order spin-phonon coupling coefficients $V_{m_{s}m_{s}^{\prime }}^{l}$ from the first-order spin-phonon interaction ${\hat{V}}^{\left(1\right)}=\sum_{lm_{s}m_{s}^{\prime }}V_{m_{s}m_{s}^{\prime }}^{l}\left(a_{l}^{†}+a_{l}\right)$ and the second-order spin-phonon coupling coefficients ${\hat{V}}^{\left(2\right)}=\sum_{ll^{\prime }m_{s}m_{s}^{\prime }}V_{m_{s}m_{s}^{\prime }}^{ll^{\prime }}\left(a_{l}^{†}+a_{l}\right)\left(a_{l^{\prime }}^{†}+a_{l^{\prime }}\right)$ from Eq. (41). The equations involve Dirac *δ* functions for conserving the energy in the process. However, finite size DFT supercell calculations do not produce continuous phonon density of states, so it is required to use a Gaussian convolution for $V_{m_{s}m_{s}^{\prime }}^{l}$ and $V_{m_{s}m_{s}^{\prime }}^{ll^{\prime }}$ which results in first-order $F_{m_{s}m_{s}^{\prime }}^{\left(1\right)}\left(\hbar \omega \right)=\sum_{l}|V_{m_{s}m_{s}^{\prime }}^{l}{|}^{2}\delta \left(\hbar \omega -\hbar \omega_{l}\right)$ and second-order $F_{m_{s}m_{s}^{\prime }}^{\left(2\right)}\left(\hbar \omega ,\hbar \omega^{\prime }\right)=\sum_{ll^{\prime }}|V_{m_{s}m_{s}^{\prime }}^{ll^{\prime }}{|}^{2}\delta \left(\hbar \omega -\hbar \omega_{l}\right)\delta \left(\hbar \omega^{\prime }-\hbar \omega_{l^{\prime }}\right)$ spectral functions in the continuum limit, respectively [156].

Finally, the appropriate spin relaxation rates are

(42) $$\begin{array}{ll} \Gamma_{2\left(m_{s}m_{s}^{\prime }\right)}^{\left(1\right)}\left(T\right) & =\frac{4\pi }{\hbar }\underset{m_{s}^{\prime \prime }}{\sum }\overset{\infty }{\underset{0}{\int }}d\left(\hbar \omega \right)n_{\text{B}}\left(\omega \right)\left[n_{\text{B}}\left(\omega \right)+1\right]\\ & \;\frac{F_{m_{s}m_{s}^{\prime \prime }}^{\left(1\right)}\left(\hbar \omega \right)F_{m_{s}^{\prime \prime }m_{s}^{\prime }}^{\left(1\right)}\left(\hbar \omega \right)}{{\left(\hbar \omega \right)}^{2}} \end{array}$$

and

(43) $$\begin{array}{ll} \Gamma_{1\left(m_{s}m_{s}^{\prime }\right)}^{\left(2\right)}\left(T\right) & =\frac{4\pi }{\hbar }\overset{\infty }{\underset{0}{\int }}\overset{\infty }{\underset{0}{\int }}d\left(\hbar \omega \right)d\left(\hbar \omega^{\prime }\right)n_{\text{B}}\left(\omega \right)\\ & \;\left[n_{\text{B}}\left(\omega^{\prime }\right)+1\right]F_{m_{s}m_{s}^{\prime }}^{\left(2\right)}\left(\hbar \omega ,\hbar \omega^{\prime }\right)\delta \\ & \;\left(\hbar \omega^{\prime }-\hbar \omega \right)\text{,} \end{array}$$

respectively. The temperature dependence enter via the Bose–Einstein occupation function (*n*_B_) of the phonons at *ω*, *ω*′ energies. In the current implementation [156], *ℏω*′ = *ℏω* constraint was employed in Eq. (43) that also enforces *l* = *l*′ and so $V_{m_{s}m_{s}^{\prime }}^{ll}$ diagonal matrix elements were considered in the reported *ab initio* calculations [156].

The numerical *ab initio* calculations provided very slow rates for $\Gamma_{2\left(m_{s}m_{s}^{\prime }\right)}^{\left(1\right)}$. The basic reason behind this observation is that *ℏω*)^2^ in the denominator completely suppresses $F_{m_{s}m_{s}^{\prime \prime }}^{\left(1\right)}$ because the largest values of $F_{m_{s}m_{s}^{\prime \prime }}^{\left(1\right)}$ are typical in the range of 1–10 MHz whereas the relevant phonon frequencies are in the order of 10 THz. This can be rationalised by noting that the first-order interaction strength is roughly *D*Δ*u*/*a*, where *D* is the zero field splitting (2.8 GHz), Δ*u* is the atomic displacement, and *a* is the nearest neighbor distance in diamond. In contrast, contributions to Raman scattering from second-order interactions scale quadratically with the second-order interaction strength, approximately *D*(Δ*u*/*a*)^2^. Thus the ratio between the first-order and second-order contributions is on the order of (*D*/*ℏω*)^2^ ∼ 10^−8^, indicating that Raman scattering due to first-order interactions can be neglected for the diamond NV centre and other *S* = 1 defects in diamond [31].

**Figure 8:**
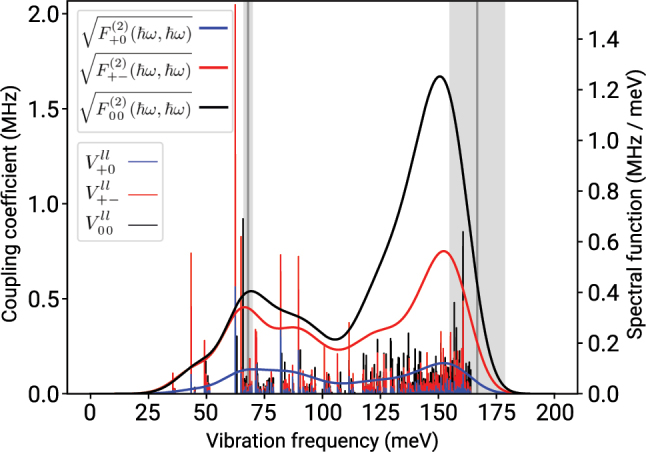
*Ab initio* calculation of the second-order spin-phonon coupling coefficients (thin lines) and spectral function (thick lines) for a single diamond NV centre in a 512-atom supercell. NV spin-phonon dynamics are characterised by the magnitudes of the matrix elements ${\hat{S}}_{z}{\hat{S}}_{+}$ (blue), ${\hat{S}}_{+}^{2}$ (red), and ${\hat{S}}_{z}^{2}-\frac{1}{3}{\hat{S}}^{2}$ (black), which cause single-flip relaxation, double-flip relaxation, and dephasing respectively. The spectral function display peaks near the values of 68.2(17) and 167(12) meV extracted from the fit of the two-phonon Orbach-process model to the experimental data (gray lines and ± 1*σ* intervals, see text).

**Figure 9:**
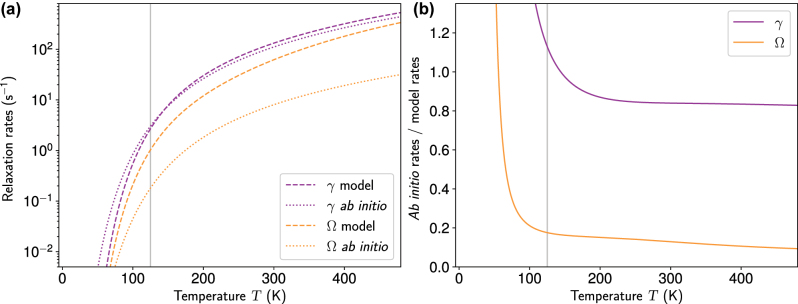
Comparison between *ab initio* theory and experiment. (a) Dotted lines show relaxation rates obtained by evaluating Eq. (43) with the *ab initio* second-order spin-phonon spectral function shown in Figure 8. Dashed lines show fit of the analytical model (see text) to the experimental data with sample-dependent constants set to zero. (b) Ratio of the *ab initio* relaxation rates to the analytical model rates. In the phonon-limited regime (gray line) the *ab initio* theory underestimates the experimentally measured relaxation rates by approximately 16% for *γ* and a factor of 8 for Ω at room temperature.

The calculated second-order spin-phonon coupling coefficients are depicted in Figure 8. Two broad peaks can be observed at certain phonon frequencies that are associated with the motion of the carbon dangling bonds so the spin density (see Ref. [156] and references therein). As the frequencies of the effective phonons are much higher than the thermal energy of the measurement temperature this will scale as Orbach-process. *Ab initio* simulation revealed that two effective phonon frequencies exist, thus Γ = 1/*T*_1_ can be described as a *double* Orbach-process, where the higher effective frequency plays a role at elevated temperatures [156]. The theory also well describes the double-flip transition and the appropriate rate equations, and both processes are double Orbach-processes. The double Orbach-process parameters could be well fitted to the experimental data with providing 68.2 meV and 167 meV effective phonon frequencies (gray lines in Figure 9) which agree well with the features of the calculated spin-phonon spectral functions. Insights from theory provided a physically well motivated model for the spin-lattice relaxation times of diamond NV centre. The calculated rates are depicted in Figure 9. The agreement between theory and experiment for *γ* is very good whereas a larger discrepancy is observed for Ω. It was hypothesized that the discrepancy in the predicted Ω is due to the exclusion of combinations of modes for which *l* ≠ *l*′, as combinations of modes with different symmetries likely account for significant matrix elements associated with pairs of different spin operators, which correspond to the single-quantum transitions. It was also discussed that $F_{00}^{\left(2\right)}\left(\hbar \omega ,\hbar \omega \right)$ in Figure 8 plays an important role in the phonon-assisted decoherence process, also showing a double Orbach-process character, which has not yet been recognized in previous works [156]. This will be discussed in the next chapter.

#### 3.5.2 Temperature shifts of magneto-optical parameters

Understanding the temperature shifts of magneto-optical parameters of defect qubits is of high importance in various aspects. One of the most obvious issues is the temperature sensing with defect qubits at the nanoscale which requires temperature characterisation of the basic magnetic parameters. Again, the diamond NV centre is the most investigated defect qubits in this regard (see Ref. [30] and references therein). As an example, the temperature dependence of the ZFS of the diamond NV centre was modelled by the thermal expansion [158] which results in an increase in the distance between carbon dangling bonds so the decrease in the ZFS parameter (*D*-constant). However, the obtained coupling coefficient was much lower than the experimental data. Recently, Tang and co-workers pointed out [159] that the thermal expansion model covers a “third order” effect as the measured magneto-optical entity *ν* will be a statistical average of the phonon mode distribution as

(44) $$〈\nu 〉=〈\nu_{0}〉+\underset{i}{\sum }\left[{\left(\frac{\partial \nu }{\partial Q_{i}}\right)}_{0}〈Q_{i}〉+\frac{1}{2}{\left(\frac{\partial^{2}\nu }{\partial Q_{i}^{2}}\right)}_{0}〈Q_{i}^{2}〉\right]\text{,}$$

where {*Q*_*i*_} are the normal coordinates of the phonons wihtin quasi-harmonic approximation. In the Born-Oppenheimer approximation, the global energy minimum results in ⟨*Q*_*i*_⟩ = 0 (forces are zero) and it becomes non-zero because of violating of the harmonic approximation, i.e., anharmonicity of the phonons. This can be taken into account in the thermal expansion of the lattice. However, the second term is then expected to be dominating. As $\left\langle Q_{i}^{2}\right\rangle =\frac{\hbar }{M_{i}\omega_{i}}\left(n_{\text{B}}\left(\omega_{i}\right)+\frac{1}{2}\right)$ with *n*_B_ Bose–Einstein occupation function Eq. (44) can be expressed as

(45) $$\left\langle \nu \right\rangle =\nu_{0}\left(a\right)+\underset{i}{\sum }\frac{1}{2}\frac{\partial^{2}\nu }{\partial Q_{i}^{2}}\frac{\hbar }{M_{i}\omega_{i}}\left(n_{\text{B}}\left(\omega_{i}\right)+\frac{1}{2}\right)\text{,}$$

where *M*_*i*_ is the mode-specific effective mass and *ν*_0_ is calculated at the lattice constant *a* which corresponds to the thermal expansion at the given temperature. The spectral function was defined as

(46) $$S_{i}\left(\omega \right)=\underset{j}{\sum }\frac{1}{M_{j}}\frac{\partial^{2}\nu }{\partial Q_{j}^{2}}\delta \left(\omega -\omega_{i}\right)\text{.}$$

The second derivative was calculated numerically as implemented in VASP in a 128-atom face centred cubic diamond supercell with 3 × 3 Monkhorst–Pack k-point sampling and DFT PBE functional [159]. The theory was applied the *D*-constant, the hyperfine constant *A*_*zz*_ and the quadrupole moment *Q*_*zz*_ of the ^14^N, and the ZPL energy of the diamond NV centre [159]. It was found that temperature dependence of the *D*-constant could be well reproduced where the dynamical effects play a major role, although, the termal expansion effect cannot be neglected. The ZPL energy shifts were well reproduced too by this theory. However, it is unexpected that the calculated spectral function for *D*-constant does not show up a peak at around 70 meV phonon energy in the study of Tang and co-workers [159], which is quite visible in the $F_{00}^{\left(2\right)}$ spectral function in Figure 8 from Thiering and Gali in Ref. [156]. The temperature shift of *Q*_*zz*_ is also well-reproduced (see Ref. [159] and references therein) and good agreement can be anticipated for *A*_*zz*_ in comparison to the few experimental data points available to date. All-in-all, this theory seems to be highly promising with good predictive power after achieving the convergent parameters in the *ab initio* simulations.

### 3.6 *Ab initio* theory of coherence of defect spins in solids

The coherence time of the defect qubits’ spin corresponds to a decoherence of the transverse nuclear spin magnetisation which is generally labelled by *T*_2_. It is also called spin-spin relaxation time as usually the interaction of the defect qubit’s electron spin with the nuclear spin bath limits its value in high quality (small electron spin bath) materials. These nuclear spins do not precess with the same frequency in real materials which can lead to a distribution of resonance frequencies around the ideal. Over time, this distribution can lead to a dispersion of the tight distribution of magnetic spin vectors, and loss of signal. This is called the dephasing time, labelled as $T_{2}^{*}$, which is associated with static or slowly varying inhomogeneities in a spin system. $T_{2}^{*}$ is the characteristic decay time of a free-induction-decay (FID) measurement, wherein a series of Ramsey sequencies (*π*/2 pulse – *τ* – *π*/2 pulse) are performed with varying free-precession interval *τ*, and an exponential decay is observed. Dephasing from fields that are static over the measurement duration can be reversed by application of a *π* pulse halfway through the free-precession interval invented by Erwin L. Hahn [160]. In this protocol, the *π* pulse alters the direction of spin precession, such that the phase accumulated due to static fields during the second half of the sequence cancels the phase from the first half. The spin phase is then refocused which appears as an echo signal in the spin resonance spectrum, i.e., Hahn-echo signal. The decay of this echo signal, due to magnetic fields that fluctuate over the course of the measurement sequence, is characterized by the coherence time *T*_2_. In practice, *T*_2_ exceeds $T_{2}^{*}$ by orders of magnitude. We further note that the phonon induced spin relaxation, longitudonal relaxation time *T*_1_, as an incoherent process places hard limits on the maximum achievable coherence times, where the theoretical limit is *T*_2_ = 2*T*_1_.

The calculation of the hard limit of *T*_2_ at a given temperature and electon/nuclear spin bath requires the calculation of *T*_1_ as described in the previous chapter. For *S* = 1 defect qubit’s spin, the |±1⟩ spin states may split due to the low-symmetry of the defect or an external small constant magnetic field. In recent studies [31, 156] it has been found for the diamond NV centre that the double-flip transition (*γ* rate) is even faster than single-flip transition (Ω rate) induced by phonons. However, *γ* was neglected in previous studies (see Ref. [156] and references therein), thus *T*_2_ have likely been overestimates which should be rewritten as

(47) $$T_{2\text{,max}}^{\left(\text{SF}\right)}=\frac{2}{3\Omega +\gamma }$$

for a superposition in the {|0⟩, |±1⟩} single-flip subspace and

(48) $$T_{2\text{,max}}^{\left(\text{DF}\right)}=\frac{1}{\Omega +\gamma }.$$

for a superposition in the {|−1⟩, |+1⟩} double-flip subspace [161, 162].

If spin-lattice relaxation does not interfere then the central spin (qubit’s electron spin) and the (nuclear) spin bath interaction and their dynamics should be simulated for obtaining the spin dephasing and decoherence times where the simulations should consider how the qubit’s spin is controlled and driven for yielding $T_{2}^{*}$ and *T*_2_ times. This is a highly complex process because the qubit’s control starts with the initialisation of the qubit, i.e., spin-polarisation of the qubit’s electron spin and the spin-polarisation may be transferred to the nuclear spin bath depending on the qubit control protocol. At certain magnetic fields, some nuclear spin levels could drive into resonance to the electron spin levels, e.g., GSLAC condition, which can significantly change the dynamics of the defect qubit’s electron spin coupled to the spin bath.

For defect qubits, the CCE approach has been successfully applied that was originally invented by Yang and Liu [144, 145]. It was originally applied to calculating the pure dephasing of the diamond NV centre’s electron spin in the large detuning regime. However, the central spin flip must be considered when the energy relaxation of the diamond NV centre is involved in the nearly-resonant regime, i.e., GSLAC condition [146]. Often, this is called generalised CCE or gCCE approach. To briefly sketch the problem and the neccessity of approximations, an open system S that consists of a central spin *s*_0_ and a number of bath spins *s*_*i*_, where *i* = {1 … *n*}. The master equation of the open system S to obtain the density matrix *ρ* can be written as

(49) $${\dot{\rho }}_{S}=-\frac{i}{\hbar }\left[H_{0},\rho_{S}\right]+E\left(\rho_{S}\right)\text{,}$$

where the Hamiltonian *H*_0_ can be written as

(50) $$H_{0}=h_{0}+\overset{n}{\underset{i=1}{\sum }}\left(h_{i}+h_{0i}\right)\text{,}$$

where *h*_0_ is the Hamiltonian of the central spin, *h*_*i*_ is the Hamiltonian of the coupled spin *s*_*i*_, and *h*_0*i*_ describes the coupling of the central spin and the bath spin *s*_*i*_. The last term on the right-hand side of Eq. (49) accounts for environmental effects that are not included in S, through the Lindbladian E that we already discussed above. One can define *h*_0_ and *h*_0*i*_ such a way which include the driving fields or other external fields like a constant magnetic field or strain field.

**Figure 10:**
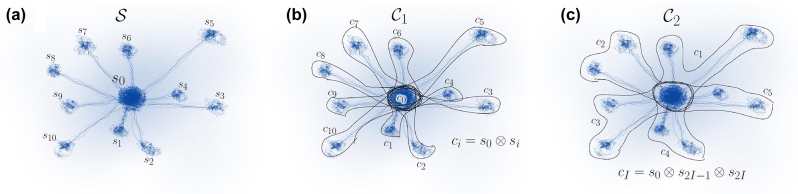
Cluster approximations of a many-spin system S. (a) S consists of a central spin *s*_0_ and number of *n* coupled spins *s*_*i*_ that couple only to the central spin *s*_0_. (b) First-order cluster approximation of S (CCE1) that comprises *n* + 1 cluster systems *c*_0_ and *c*_*i*_. *c*_0_ includes the central spin *s*_0_ only, while *c*_*i*_ for *i* ≠ 0 includes a pair of spins, *s*_0_ and *s*_*i*_. (c) Second-order cluster approximation of S (CCE2) that comprises *n*/2 + 1 cluster systems *c*_*I*_, where each cluster system contains *s*_0_ and two coupled spins *s*_*I*_ where 1 ≤ *I* ≤ *n*/2. *c*_0_ includes solely the central spin *s*_0_.

The size of the problem, i.e., the dimension of the Hilbert space, increases exponentially with *n*, which makes an exact solution unfeasible for large *n*. To model the dynamics of S it is divided into a cluster $C_{N}$ of overlapping cluster systems, where N is the order of the cluster approximation as illustrated in Figure 10. All of the subsystems are artificially coupled then together through a modified Lindbladian superoperator

(51) $$\begin{array}{ll} L\left(b_{il}\right) & =\underset{l}{\sum }\frac{{\dot{b}}_{il}}{\mathrm{Tr}\left(C_{il}^{†}C_{il}\rho_{c_{i}}\right)}\\ & \;\times \left(C_{il}\rho_{c_{i}}C_{il}^{†}-\frac{1}{2}\left(\rho_{c_{i}}C_{il}C_{il}^{†}+C_{il}^{†}C_{il}\rho_{c_{i}}\right)\right) \end{array}$$

added to the master equation of each subsystem, where and *C*_0*l*_ and *C*_*il*_ are Lindblad operators. We consider *C*_0*l*_ and *C*_*il*_ operators that describe solely spin flip-flop transitions of the central spin. Here *b*_*il*_ are time-dependent rates determined from the flip-flops occurring in subsystem other than *i*. The Lindbladian formalism ensures that all the spin flip-flops occurring in the different subsystems is carried out in all subsystems. This way the central qubit replicas evolve identically in all subsystems. Due to the extended Lindbladian, spin momentum is conserved no longer in the subsystems but in the whole cluster approximation. This way the cluster approximation together with the Lindbladian coupling describes the dynamics of the whole qubit-spin bath system approximately. Considering the dynamics, the main approximation of the method is the neglect of the intra spin bath coupling and entanglement that may affect the dynamics of the central qubit through spin diffusion as well as constructive and destructive interference that can give rise to echo signals and dark states, respectively. These limitations can, however, be systematically lifted by including more and more environmental spins in the subsystems of the cluster approximation.

In the CCE approach, the corresponding Hilbert-space can be significantly truncated that are coupled to each other in which the density matrix of the central spin can be consistently calculated. As we increase the order of expansion, the results should converge to the theoretical limit, in good analog to the CI expansion method for approaching the accurate correlation energy of the many-electron system. For instance, Yang and co-workers found for the diamond NV centre in the nuclear spin bath of remote ^13^C nuclear spins [146] that gCCE4 and gCCE5 results agree, thus gCCE4 can considered as absolutely convergent for this particular system.

Seo and co-workers [163] applied CCE method for diamond NV centre and divacancy qubits in 4H SiC for understanding the spin dynamics between the qubit’s electron spin and the nuclear spin bath with assuming natural abundance of ^13^C at 1.1% and ^29^Si at 4.5%. The authors ignored the Fermi-contact term in the hyperfine interaction between the electron spin and the nuclear spins. A constant magnetic field was applied in the simulation. We note that because not the gCCE method was applied in Ref. [163], therefore, these simulations could not well describe the spin dynamics for the magnetic fields at GSLAC and ESLAC conditions of the systems, which results in a rapid decrease of the coherence times. This was later done by gCCE method for divacancy qubits in 4H SiC [164]. Seo and co-workers found that CCE2 level of theory well converges with the afore-mentioned conditions and the radius of the spin bath at around 50 Å from the defect qubit’s spin provides convergent results. At the CCE2 level, the distance between interacting nuclear spins was set to 8 Å which converged well [163]. It was found that ensemble averages over 50 samples are good enough to produce numerically converged results. For the magnetic fields above 30 mT they found a simple relation between the $T_{2}^{*}$ times and the concentrations of ^13^C and ^29^Si isotopes in the SiC crystal showing that the ^13^C and ^29^Si nuclear spins are completely decoupled due to the different Zeeman-splitting for these two species which hinders spin flip-flop processes between these types of nuclear spins. As a consequence, the spin dephasing times of divacancy qubits in 4H SiC are equal or greater to that of diamond NV centre despite the larger density of *I* = 1/2 nuclear spins in SiC than that in diamond [163]. This conclusion was earlier achieved in a similar study applied to the Si-vacancy qubit (*S* = 3/2) in 4H SiC [165] as we quote below. For a given cut-off distance *R*_*c*_, the number of total nuclear spins (*N*_C_ + *N*_Si_) surrounding the Si-vacancy centre in SiC is about 2.6 times as large as that of the diamond NV centre, due to the larger ^29^Si abundance. The reasons for the $T_{2}^{*}$ time of Si-vacancy centres not being reduced by the same factor are as follows: (i) the C-Si bond length 1.89 Å in SiC is larger than the C–C bond length 1.54 Å in diamond, which implies the volume density of nuclear spins is reduced by a factor of (1.89/1.54)^3^ = 1.8; and (ii) about 80% of the nuclear spins in the bath are ^29^Si, which have smaller gyromagnetic ratios than ^13^C (|*γ*_C_/*γ*_Si_| ≈ 1.3) and, as a result, produce weaker hyperfine fluctuations. These two factors compensate the larger natural abundance of the ^29^Si, and results in similar $T_{2}^{*}$ times of defect qubits in SiC and NV centres in diamond.

In a subsequent publication, the spin dephasing times were calculated for hypothetical defect spins with no Fermi-contact hyperfine interaction with moderate magnetic fields (0.1–0.5 mT) in 2D materials [166] by applying the same method. The subject of this study was later extended to 12,000 materials in which both spin dephasing (free-induction decay) and spin coherence (Hahn-echo) times were considered at large constant magnetic fields (e.g., 5 T) [13]. With these simulation conditions, Seo and co-workers [166] found for hBN and molybdenum-disuplhide (MoS_2_) materials that the spin dephasing time in bulk hBN should be around 18 μs for natural abundance of ^11^B and ^10^B isotopes whereas it is about 1.18 ms in MoS_2_. They attributed the orders of magnitude difference to partially to the variant of nuclear spin density in the two materials and partially to the relatively small gyromagnetic constant of Mo isotopes [166]. By replacing all ^11^B by ^10^B should result in >2 factor improvement in the spin dephasing time in hBN, according to the model. Kanai and co-workers [13] concluded that SiC and Si could reach *T*_2_ ≈ 5 ms beating even diamond (≈4 ms). They identified various chalgogenides which have very long intrinsic spin-spin related *T*_2_ times at magnetic field of 5 T such as CeO_2_ (≈179 ms), CaO (≈77 ms), *α*-quartz (≈8.5 ms), wurtzite ZnO (≈4.2 ms), and MgO (≈1.33 ms). It should be mentioned that the $T_{2}^{*}$ time can be long in a common 2D material, WS_2_ (≈11 ms). These results should be interpreted with the caveat that the temperature-dependent spin-lattice relaxation times and other limiting factors are not included in that study, thus these results might be valid at relatively low operation temperatures for many materials.

Although, the results on spin dephasing and coherence times with hypothetical defects are somewhat indicative for classifying materials, the defect qubit’s spin relaxation properties may crucially depend on the *local* environment induced by the defect in terms of ZFS, strain fields and spin density distribution. The last entity is in particular important for materials with dense nuclear spin bath. In that case, the Fermi-contact term in the hyperfine tensor is dominant, and such an effect cannot be fully neglected even in diamond or SiC with relatively dilute nuclear spin densities.

For *S* = 1 defects the case of GSLAC or ESLAC condition was already mentioned where a simple external parameter, magnitude and direction of the constant magnetic field, may drastically change the coherence properties of the defect qubits, e.g., the interplay between the actual *D*-constant of the defect spin and the strength of the constant magnetic field [62, 147, 148, 164]. Another interesting example is the so-called clock-transition quantum optics protocol which may be realized by low-symmetry defect spins, e.g., basal divacancy defects in 4H SiC, where the |+1_*z*_⟩ and |−1_*z*_⟩ levels naturally splits (see Refs. [164, 167] and references therein). Combining the *E* = 18.4 MHz splitting with a strong longitudinal splitting (*D* = 1.334 GHz), the ZFS tensor leads to an avoided crossing of electron spin levels at zero magnetic field from which a clock transition emerges. The qubit levels at the clock transition correspond to $|+〉=\left(1/\sqrt{2}\right)\left(|1_{z}〉+|-1_{z}〉\right)$ and |0⟩ = |0_*z*_⟩ (e.g., see Ref. [167]). The frequency of clock transitions is insensitive to magnetic fields to first order, thus increasing protection from the nuclear bath induced decoherence. Onizhuk and co-workers proved by gCCE theory that the clock transition can indeed elongate the coherence times and the coherence times can be further elongated with opening the gap between |+1⟩ and |−1⟩ levels which indeed occurs for the other basal plane divacancy qubit with *E* = 82.0 MHz and *D* = 1.222 GHz ZFS parameters [164]. In experiments, the fluctuating electric fields can lead to a serious decoherence for |+⟩ state, so the charges should be depleted to observe the predicted improvement in the coherence times that was achieved by applying an external electric field to the system [164, 168]. In the simulations, the fully convergent results were achieved at gCCE4 level.

Another important defect spin is the negatively charged boron-vacancy ($V_{\text{B}}^{-}$) in hBN that was already mentioned in our review paper. The defect has three nitrogen dangling bonds with large (≈47 MHz) hyperfine coupling to the electron spin (*S* = 1) with ZFS in the GHz region both for the ground and excited states (see Ref. [27] and references therein). In other words, the three ^14^N nuclear spins are strongly coupled to the electron spin for which the Fermi-contact term is dominant. This explains a recent observation [24] that the spin coherence time of $V_{\text{B}}^{-}$ in hBN is much shorter at the condition of close to zero magnetic fields than that previously anticipated, and it is below 0.1 μs, c.f., ≈18 μs in Ref. [166] for a hypothetical defect. Ivády and Gali carried out gCCE simulations on this system at the experimental external magnetic field of 14 mT^24^. It was found that gCCE3 level is convergent. The maximum distance between the defect spin and the nuclear spin should be around 10 Å whereas the maximum distance between the nuclear spins should be around 7.5 Å, in order to achieve convergent results. The simulated spin echo decay curves of the centre in h^11^BN and h^10^BN are fitted with a stretched exponential function, $\exp\left[-{\left(t/T_{2}\right)}^{n}\right]$, leading to *T*_2_ = 92 ns and *T*_2_ = 115 ns for h^11^BN and h^10^BN, respectively, with an exponent *n* ≈ 1.35. The dependence of the coherence time with the isotopic content exhibits a linear increase with the ^10^B abundance. This effect results from the reduced nuclear gyromagnetic ratio of ^10^B that weakens the hyperfine interaction and the boron nuclear spin flip-flop rate, both of which has a positive impact on the coherence time of the central spin. In a recent study it has been shown [169] that the long *T*_2_ ≈ 27 μs can be retained for $V_{\text{B}}^{-}$ in hBN at giant external magnetic fields, e.g., 3 T, which suppresses the strong electron-nuclear spin couplings.

At moderate external magnetic fields, it is challenging to observe the Rabi-oscillation of the $V_{\text{B}}^{-}$ electron spin because of the intrinsically short coherence times. Ivády and Gali showed by gCCE simulations [25] that if the microwave field is tuned at the centre hyperfine peak or the *m*_*I*_ = ±1 hyperfine peaks of the strongly coupled three ^14^N spins then the Rabi-oscillation of the 4-spin $V_{\text{B}}^{-}$ becomes observable, and even 10 MHz detuning significantly suppresses the amplitude of the Rabi-oscillation. In experiments [25], one can see a multiple-frequency oscillation, in which a beat is clearly recognized, and it is superposed on another slow oscillation. The results could be interpreted by the results from gCCE simulations: the nearest neighbour ^14^N nuclear spins are driven by the microwave field at the given magnetic field (44 mT) and the observed multiple frequencies in Ramsey interference correspond to spin-rotation frequencies in rotating frame on three hyperfine levels. The three frequencies in the spectrum can be identified as the detuning between the microwave field and the centre hyperfine level, as well as the *m*_*I*_ = ±1 levels [25]. The spin bath of the 4-spin $V_{\text{B}}^{-}$ system was further simulated by taking 127 ^14^N and 127 ^11^B nuclear spins into account in which the HSE06 hyperfine tensor and electric field gradient tensors were applied. In the simulations, an effective spin-polarisation transfer could be observed towards these ^14^N nuclear spins whereas the spin-polarisation towards ^11^B is small. Hence, it was concluded that the neighbour ^14^N nuclear spins are responsible for the modulation of the Rabi oscillation, including the decay of the background beyond 0.2 μs, and rest of the spin bath is responsible for the decay of the Rabi oscillation [25].

In pulsed electron spin resonance measurements, the 4-spin nature of $V_{\text{B}}^{-}$ was further confirmed [26]. The measurements were carried out in a W-band (94 GHz) microwave resonator which brings the electron spin resonance frequencies at around 3.5 T. At this high magnetic fields, the *T*_2_ time of $V_{\text{B}}^{-}$ extends to 15.1 μs observed by electron spin echo (ESE) measurements [26], in good agreement with the recent gCCE3 simulations [169]. The decay curve reveals its oscillatory behavior especially pronounced at the very beginning of the transient curve. Such oscillations refer to electron spin echo envelop modulation (ESEEM) and manifest the presence of coherent coupling of the $V_{\text{B}}^{-}$ electron spin with magnetic moments of nuclei available in the hBN lattice. The observed beating frequencies corresponds to the nuclear magnetic resonance frequencies (consisting of the combination of hyperfine and quadrupole interactions) of the three nearest neighbour ^14^N nuclear spins. PBE DFT calculations confirmed that no other ^14^N near the vacancy could produce such nuclear spin resonances [26] which makes the 4-spin $V_{\text{B}}^{-}$ model consistent. Finally, it was found that the optical nuclear spin-polarisation at the GSLAC and ESLAC conditions of the external magnetic fields at respective 124 mT and 74 mT can be efficiently carried out towards the neighbour ^14^N nuclear spins and it can be coherently driven at ≈5 MHz which is much faster than the appropriate nuclear spin resonance frequency [27]. To our knowledge, the spin dephasing and spin-echo simulations for these strongly coupled systems have not yet been reported.

## 4 Summary

In this paper, we reviewed the recent advances on *ab initio* theory on defect qubits. A strong emphasis was put on the calculation of excited states, photo-ionisation thresholds and optical excitation spectra also as a function of temperature. A novel theory has been developed on the effective mass states of the excited states of deep defects. Major breakthroughs have been presented on the calculation spin dynamics of the defect qubits which converted the phenomenological description of the spin relaxation times to fully *ab initio* solution.
